# The state of oligomerization of Rubisco controls the rate of synthesis of the Rubisco large subunit in *Chlamydomonas reinhardtii*

**DOI:** 10.1093/plcell/koab061

**Published:** 2021-02-24

**Authors:** Wojciech Wietrzynski, Eleonora Traverso, Francis-André Wollman, Katia Wostrikoff

**Affiliations:** 1 Sorbonne Université, CNRS, Institut de Biologie Physico-Chimique, Unité Mixte de Recherche 7141, 75005 Paris, France; 2 Department of Molecular Structural Biology, Max Planck Institute of Biochemistry, 82152 Martinsried, Germany; 3 Helmholtz Pioneer Campus, Helmholtz Zentrum München, 85764 Neuherberg, Germany

## Abstract

Ribulose 1,5-bisphosphate carboxylase/oxygenase (Rubisco) is present in all photosynthetic organisms and is a key enzyme for photosynthesis-driven life on Earth. Its most prominent form is a hetero-oligomer in which small subunits (SSU) stabilize the core of the enzyme built from large subunits (LSU), yielding, after a chaperone-assisted multistep assembly process, an LSU_8_SSU_8_ hexadecameric holoenzyme. Here we use *Chlamydomonas reinhardtii* and a combination of site-directed mutants to dissect the multistep biogenesis pathway of Rubisco in vivo. We identify assembly intermediates, in two of which LSU are associated with the RAF1 chaperone. Using genetic and biochemical approaches we further unravel a major regulation process during Rubisco biogenesis, in which LSU translation is controlled by its ability to assemble with the SSU, via the mechanism of control by epistasy of synthesis (CES). Altogether this leads us to propose a model whereby the last assembly intermediate, an LSU_8_-RAF1 complex, provides the platform for SSU binding to form the Rubisco enzyme, and when SSU is not available, converts to a key regulatory form that exerts negative feedback on the initiation of LSU translation.

## Teaser

A Rubisco assembly intermediate is involved in repressing synthesis of unassembled Rubisco large subunit in Chlamydomonas.

## Introduction

Ribulose bisphosphate carboxylase/oxygenase (Rubisco) is the key enzyme in the light-driven carbon assimilation pathway and is present in all photosynthetic organisms. Emerging around 3.5 billion years ago, even before the beginning of oxygen-evolving photosynthesis, it is one of the most abundant proteins on Earth (Ellis, 1979; Tabita et al., 2008; Bar-On and Milo, 2019). Operating as the first step in the Calvin–Benson–Bassham cycle, Rubisco catalyzes the fixation of atmospheric CO_2_ into biologically available organic carbon. Throughout time, Rubisco evolved into numerous different forms (Andersson and Backlund, 2008; Tabita et al., 2008). The most widespread clade, form I, consists of Rubisco formed by both large (LSU) (∼52* *kDa) and small (SSU) (∼16* *kDa) subunits. Rubisco form IB, a further subclade division, is present in cyanobacteria as well as in green algae and plants (Hauser et al., 2015). In the latter eukaryotic organisms, the two subunits are encoded by spatially separated genomes of different ploidy. LSU is encoded by a single gene (*rbcL*) in the highly polyploid chloroplast, whereas SSU is a product of a family of nuclear genes (*RBCS*). Both subunits assemble in a 1:1 ratio in the chloroplast stroma, to create a hexadecameric holoenzyme (Andersson and Backlund, 2008).

**Figure:**
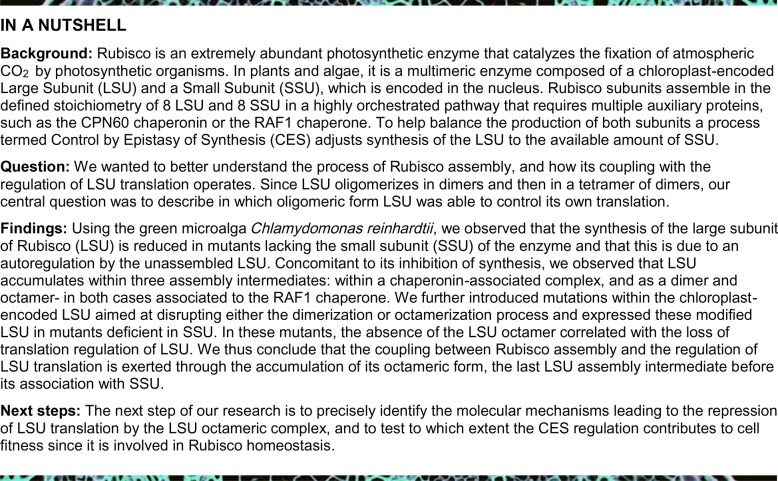


Recently, significant progress was made in our understanding of the mechanisms leading to Rubisco biogenesis in the chloroplast, which rely on conserved features of cyanobacterial Rubisco assembly that also exhibit eukaryotic specificities (reviewed in Bracher et al., 2017; Vitlin Gruber and Feiz, 2018). As mentioned above, a eukaryotic feature of green algae and vascular plants consists in the transfer of *RBCS* genes to the nucleus, no longer clustered with the *rbcL* gene in an operon, which allows for further regulatory processes to take place. *RBCS* genes were indeed early characterized as being part of the PhANG gene family (reviewed in Chan et al., 2016; Börner, 2017), a set of genes undergoing retrograde signaling in response to chloroplast translation and redox status. Once translated in the cytosol, SSU is translocated into the chloroplast via the Tic/Toc import machinery (Jarvis, 2008) where it undergoes cleavage of its targeting peptide and post translational Met1 modification (Grimm et al., 1997). Then, it may interact with the chaperone Rubisco accumulation factor 2 (RAF2), an inactive form of pterin carbinolamine dehydratase, which delivers the protein to an LSU oligomeric complex for proper assembly into the final Rubisco holoenzyme (Feiz et al., 2014), based on RAF2 and SSU coimmunoprecipitation and on the decreased Rubisco accumulation observed in maize mutants. The observation that Arabidopsis mutants presenting a decrease in SSU production also display reduced accumulation of RAF2 offers a further hint that RAF2 has a role in SSU chaperoning (Fristedt et al., 2018).

LSU biogenesis on the other hand starts with the expression of the chloroplast *rbcL* gene, which relies on the eukaryote-specific factor MRL1. MRL1, a nuclear-encoded organellar transacting factor belonging to the pentatricopeptide repeat protein family (Woodson and Chory, 2008; Barkan and Small, 2014; Hammani et al., 2014), contributes to the stabilization of the *rbcL* transcript in Chlamydomonas and of its processed form in Arabidopsis (Johnson et al., 2010). Because of the hydrophobic nature of the LSU surface, making it aggregation-prone, proper folding of nascent LSU requires the assistance of molecular chaperones. Based on a model derived from bacterial studies (Langer et al., 1992; Hartl, 1996), it has been suggested that newly synthesized LSU is recruited by the chloroplast folding machinery, interacting first with HSP70B/CDJs/CGE1, the plastid homologs of the DnaK/DnaJ/GrpE chaperones (Willmund et al., 2008) and subsequently with the CPN60/CPN20/CPN23/CPN10 chaperonin complex (Brutnell et al., 1999). However, despite the requirement for the DnaK/DnaJ/GrpE chaperones in the recombinant expression of Rubisco in bacteria (Checa and Viale, 1997), there is no in vivo experimental evidence that LSU expressed in the chloroplast is indeed a client protein for these factors, which may suggest that neosynthesized LSU is directly captured by the chaperonin complex. LSU would then oligomerize in a step-wise manner to create an octameric core of the enzyme with the help of other assembly chaperones. Two of these chaperones, RBCX (Onizuka et al., 2004) and RAF1 (Feiz et al., 2012), are of cyanobacterial origin where they have been shown to stabilize LSU during dimerization and further oligomerization until the binding of SSU, thereby leading to a displacement of the chaperones as demonstrated in vitro (Liu et al., 2010; Bracher et al., 2011; Hauser et al., 2015; Kolesinski et al., 2017). Although RBCX and RAF1 were shown to promote folding and assembly of the LSU_8_ core in vitro, experimental evidence to support their role in LSU oligomerization in vivo is lacking, even though LSU can be identified as a major interactant for both chaperones. Indeed, LSU co-immunoprecipitates with Strep-tagged RBCXs from Arabidopsis extracts, indicating that RBCX may bind to plant LSU as well (Kolesinski et al., 2011). Similarly, LSU is co-immunoprecipitated with RAF1 in maize extracts (Feiz et al., 2012) or captured from cyanobacterial extracts when mixed with a recombinant RAF1 Strep-tagged variant (Kolesinski et al., 2014, 2017).

Whether RAF1 and RBCX are functionally redundant is still under debate. RBCX is dispensable in vivo, at least in the β-cyanobacterial species in which this gene does not cluster within the Rubisco operon, such as in *Synechococcus elongatus* sp. PCC7942 (Emlyn-Jones et al., 2006). Whether this dispensability still holds true for cyanobacterial species harboring a Rubisco LXS operon awaits further confirmation (Onizuka et al., 2004; Emlyn-Jones et al., 2006; Tarnawski et al., 2008). To date, there is no evidence of its requirement in algae and plants, where two RBCX isoforms, which would form homodimers, have been described (Kolesinski et al., 2013; Bracher et al., 2015). The requirement for RAF1 also varies between species: a RAF1 knockout is lethal for maize seedlings and results in Rubisco deficiency (Feiz et al., 2012). Moreover a cognate RAF1 is required for plant Rubisco assembly, indicating a tight LSU-RAF1 coevolution (Whitney et al., 2015). In sharp contrast, the absence of RAF1 in cyanobacteria still allows Rubisco formation, as monitored in *Synechocystis* sp. PCC 6803 (Kolesinski et al., 2017) and in *S. elongatus* PCC7942. In the latter case, Rubisco amount was decreased by one-third, followed by a defect in carboxysome formation, resulting in reduced cell growth in air (Huang et al., 2020). Interestingly, while both RAF1 and RBCX appear to be non-essential in cyanobacteria and seem to be able to interact in vitro with LSU oligomers of the same order, their mode of action may be different: the resolution of LSU-containing crystals (Bracher et al., 2011; Hauser et al., 2015; Huang et al., 2020; Xia et al., 2020) indicates indeed that RBCX and RAF1 have different binding sites on LSU, and may work sequentially, as RAF1 is able to displace RBCX from LSU while the converse is not true (Kolesinski et al., 2017). Second, the respective knock-outs observed in cyanobacteria exhibit different phenotypes with respect to Rubisco amount and carboxysome formation, casting doubts about their possible redundancy (Huang et al., 2019, 2020).

A second eukaryote-specific factor is required for Rubisco biogenesis, namely the bundle sheath defective 2 (BSD2) protein, first identified in maize (Brutnell et al., 1999). While the role of BSD2 remained for a long time hypothetical, a breakthrough study highlighted the requirement for the BSD2 chaperone for higher plant Rubisco recombinant production in order to stabilize LSU at the final stage of Rubisco formation before SSU binding (Aigner et al., 2017). In addition, in vivo data indicates that tobacco BSD2 comigrates with Rubisco (Conlan et al., 2019) suggesting that a BSD2-LSU complex is the end-state intermediate in plants. Additional roles for BSD2 in chloroplast coverage and/or division (Salesse et al., 2017; Li et al., 2020) have been proposed in maize, but may be restricted to C4 plant lineages, as they were not observed in tobacco (Conlan et al., 2019). Last BSD2 may also participate in a repair mechanism of oxidized Rubisco (Busch et al., 2020). There is little evidence for a functional ortholog of BSD2 in green algae. In Chlamydomonas, the ZNJ2 and ZNJ6 proteins share homology with plant BSD2 that is restricted to a Zn finger domain characteristic of DNAJ-like proteins (Doron et al., 2014, 2018). There is, as yet, no report of a role for ZNJ2/ZNJ6 in Rubisco biogenesis. It is worth noting that the putative SSU-chaperone RAF2 also interacts with LSU in vivo (Feiz et al., 2014) and is required in the recombinant production of plant LSU *in Escherichia coli*, even in the absence of SSU (Aigner et al., 2017). Finally, LSU also undergoes some post-translational modifications but their occurrence along the biogenesis pathway remains to be understood (Houtz et al., 2008).

Since the two Rubisco subunits are expressed in two different compartments, there is a need for regulation to coordinate their production in the stoichiometric amounts required for their functional assembly. It has been demonstrated that other multimeric photosynthetic complexes, such as Photosystems I and II (PSI and II, respectively), cytochrome *b_6_f* (cyt *b_6_f*), and ATP-synthase undergo translation regulatory processes, known as control by epistasis of synthesis (CES), which sense their assembly state in *Chlamydomonas reinhardtii* chloroplasts (Kuras and Wollman, 1994; Choquet et al., 1998; Wostrikoff et al., 2004; Minai et al., 2006; Drapier et al., 2007) as well as in higher plant chloroplasts (Levey et al., 2014; Chotewutmontri and Barkan, 2020). CES results in an adjustment of the rate of translation of a subset of chloroplast-encoded subunits that is related to the presence of their assembly partners. Accordingly, earlier observations of Chlamydomonas* RBCS* knockout mutants showed that LSU synthesis is strongly decreased in the absence of SSU (Khrebtukova and Spreitzer, 1996). Similarly, tobacco *RBCS* knock-down lines (Rodermel et al., 1996) displayed a down-regulation of LSU synthesis. It was subsequently demonstrated that unassembled LSU exerts negative feedback on its own translation in maize and tobacco chloroplasts (Wostrikoff and Stern, 2007; Wostrikoff et al., 2012). However, the nature of the LSU assembly intermediate that controls LSU translation has not been determined. The documentation of CES behavior of the LSU in vascular plants and green algae makes this an excellent case study for the evolution of the underlying regulatory mechanism.

As a first step toward this goal, we undertook a detailed molecular characterization of the LSU assembly intermediates in Rubisco biogenesis in the genetically tractable microalgae *C. reinhardtii*. Using a series of mutants variously affected in the presence of Rubisco LSU and SSU subunits and impairing Rubisco assembly, we were able to identify the in vivo intermediates of Rubisco formation and LSU-chaperone(s) complexes. We then dissected the CES mechanism governing LSU translation in the absence of SSU and provide evidence that CES control of *rbcL* translation relies on the specific accumulation of a hetero-oligomer, comprising at least LSU and RAF1, in the absence of SSU.

## Results

### Downregulation of LSU synthesis in a Chlamydomonas RBCS mutant

**Figure 1:**
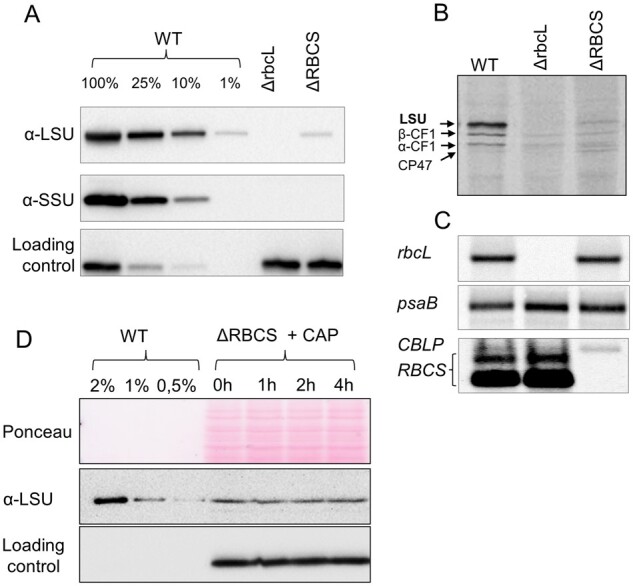
LSU accumulation, synthesis rate, and stability in the absence of its assembly partner. (A) Immunoblot showing protein accumulation of Rubisco subunits in the ΔRBCS strain, using an antibody directed against whole Rubisco holoenzyme. PsaD accumulation, revealed with a specific antibody, is shown as a loading control. (B) ^14^C pulse labeling experiment showing the synthesis rate of LSU in the ΔRBCS strain as compared to the WT in upper panel (positions of LSU as well as ATPase α and β subunits and PSII CP43 subunit are indicated by arrows). (C) mRNA accumulation in the same strains as in B, as probed by hybridization with *rbcL* and *RBCS* probes, and *psaB and CBLP* probes used as loading controls. In both panels, the Δ*rbcL* strain exhibiting a deletion of the *rbcL* gene is used as a negative control. (D) Unassembled LSU stability assayed by immunochase over 4* *h after chloroplast synthesis arrest by chloramphenicol (CAP) addition. LSU is detected with the anti-Rubisco antibody, cyt *f* is used as a loading control.

Previous work showed that deletion of the *RBCS* genes in *C. reinhardtii* resulted in a significant decrease in synthesis of LSU (Khrebtukova and Spreitzer, 1996). We confirmed this observation in an independent mutant, hereafter called ΔRBCS strain which, in contrast to one previously described, is fertile, thus allowing further genetic analysis. We isolated this strain from backcrosses between our laboratory reference strain and the Cal.005.013 strain (Dent et al., 2005), which harbors a large deletion encompassing the two *RBCS* linked genes. In the absence of SSU, LSU accumulated to ∼1% of wild type (WT) level, which argues for a concerted accumulation of Rubisco subunits (Figure 1A). Moreover, as reported by Khrebtukova and Spreitzer (1996), LSU exhibited a lower rate of synthesis in ΔRBCS compared to WT as shown by ^14^C pulse-labeling experiments (Figure 1B). This down-regulation is posttranscriptional, since *rbcL* mRNA level was not affected in the ΔRBCS strain as compared to WT (Figure 1C). To confirm that the decrease in LSU radiolabeling truly represents a decrease in translation rather than massive proteolytic degradation, we tested the stability of unassembled LSU in the ΔRBCS strain by following LSU accumulation over 4* *h in presence of chloramphenicol, an inhibitor of chloroplast protein synthesis. As shown in Figure 1D, unassembled LSU was found to be stable over this time period (see also Supplemental Figure S1).

### rbcL initiation of translation is impaired in absence of SSU

**Figure 2:**
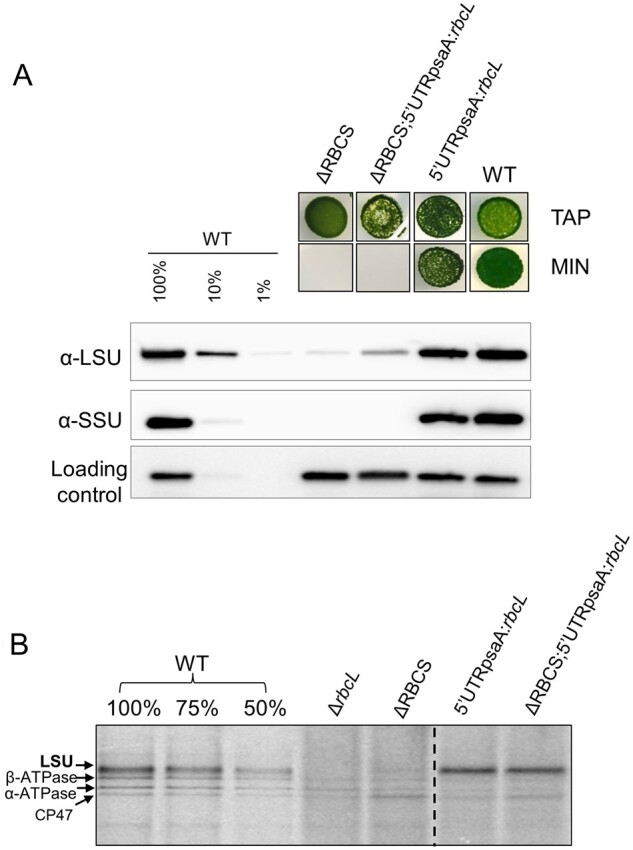
Swapping *rbcL* 5′ UTR regulatory sequence impairs the CES regulation. (A) Upper panel: Photosynthetic growth phenotypes of 5′UTRpsaA:*rbcL* strains defective or not for Rubisco SSU, and accumulation of the corresponding Rubisco subunits tested by western blot analysis. Lower panel: In ΔRBCS;5′UTRpsaA:*rbcL*, LSU is accumulating to higher levels than in ΔRBCS. TAP stands for TAP medium, MIN is an acetate-free, phototrophy-selective medium. (B) ^14^C pulse labeling experiment showing LSU synthesis rate in 5′UTRpsaA:*rbcL*-background with and without small subunit compared with wild-type, Δ*rbcL* and ΔRBCS strains. The dashed line marks the position where two irrelevant lanes were removed.

As shown previously, those subunits of photosynthetic complexes that undergo CES translational regulation bear, within the 5′UTR of their mRNA, all cis-acting elements controlling this process (Choquet et al., 1998; Choquet and Wollman, 2007). Thus, in all cases studied so far, regulation of translation of CES proteins occurs at the initiation step. To test whether the native *rbcL* 5′UTR is required for *RBCS*-sensitive down regulation of *rbcL* translation, we replaced it by the *psaA* 5′UTR. After biolistic transformation of the ΔrbcL strain (ΔR T1.3; mt+) by the 5′UTRpsaA:*rbcL* chimera (“Materials and methods”; Supplemental Table S1), we obtained phototrophic transformants which accumulated WT levels of LSU, demonstrating that *psaA* 5′UTR is able to drive *rbcL* expression efficiently (Figure 2A). We then crossed a representative 5′UTRpsaA:*rbcL* transformant (mt+) with the ΔRBCS strain (Cal.13.1B; mt−) and obtained progeny displaying uniparental inheritance of the chloroplast 5′UTRpsaA:*rbcL* chimeric gene and a 2:2 distribution of the Δ*RBCS* nuclear mutation. Progeny from distinctive genotypes (5′UTRpsaA:*rbcL* and ΔRBCS; 5′UTRpsaA:*rbcL*), easily identified by their distinct acetate requirement for growth (Figure 2A), were used in pulse labeling experiment to monitor *rbcL* translation rates in this non-native 5′UTR context. As shown in Figure 2B, the *rbcL* translation rate was now similar between sister strains, (harboring *RBCS* or not), and it proceeded at the same rate as in the WT. This experiment demonstrates that *rbcL* 5′UTR is required for CES regulation of LSU synthesis since its replacement by the upstream sequence of another chloroplast gene allows *rbcL* translation to become CES-insensitive. Yet, we note that LSU accumulation in absence of SSU is nevertheless reduced compared to the strain harboring the 5′UTRpsaA:*rbcL* chimera and expressing SSU. However, the decrease in LSU accumulation is less pronounced than when LSU is translated from the native *rbcL* gene in absence of SSU (see “Discussion” Section).

**Figure 3:**
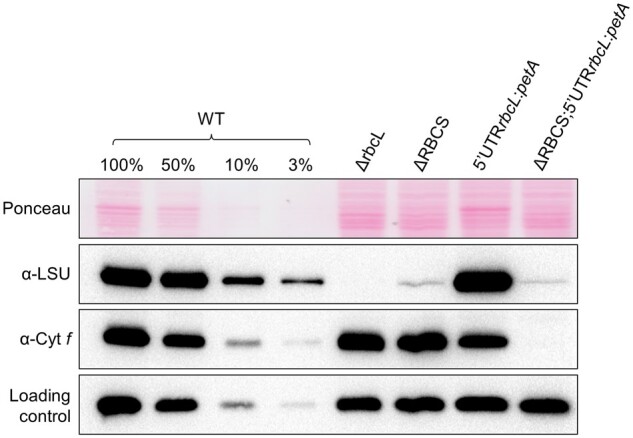
Expression of cyt *f* is inhibited in the absence of Rubisco small subunit. Immunoblot using antibodies directed against the proteins depicted at the left, showing Rubisco and cyt *f* accumulation levels in the wild-type, ΔRBCS, ΔrbcL, and 5′UTRrbcL:*petA* strains with and without SSU. PsaD accumulation is shown as a loading control.

To test whether *rbcL* 5′UTR is sufficient to confer CES regulation to an unrelated gene, we expressed a fusion between the *rbcL* 5′UTR and the *petA* gene encoding cytochrome *f* (cyt *f*), a core protein of the cyt *b*_6_*f* complex (5′UTRrbcL:*petA*; hereafter referred to as the reporter) in presence or absence of SSU (see “Materials and methods”; Supplemental Table S1). Prior research (Chen et al., 1995; Choquet et al., 1998) has shown that in normal growth conditions cyt *f* accumulation level mirrors its rate of synthesis, which makes it a faithful reporter (proxy) of translation efficiency. In the experiment shown in Figure 3, we compared the accumulation of the cyt *f* reporter protein in representative transformants in the presence (5′UTRrbcL:*petA*) or absence (ΔRBCS; 5′UTRrbcL:*petA*) of SSU. Expression of the reporter fusion driven by the *rbcL* 5′UTR in presence of SSU led to significant cyt *f* accumulation, albeit at lower levels than observed in WT. However, cyt *f* accumulation driven by the *rbcL* 5′UTR became almost undetectable in absence of SSU, thus showing the same behavior as LSU. Altogether, these observations demonstrate that the 5′UTR of the *rbcL* gene contains all information required to confer Rubisco assembly-dependent regulation of translation to a downstream coding sequence.

### Translation initiation is inhibited by unassembled LSU

Two possible mechanisms could account for the observed translational repression of *rbcL* in absence of SSU. Either the small subunit is required for direct or indirect trans-activation of LSU translation, or, in the absence of its SSU partner, unassembled LSU is inhibiting its own translation via an auto-regulatory feedback. In order to distinguish between these two hypotheses, we followed the expression of the *rbcL* 5′UTR-driven-reporter gene in a context where both Rubisco subunits are absent (as detailed in Choquet and Wollman, 2007). A trans-activation model predicts that the absence of SSU and LSU should yield a low accumulation of the reporter, similar to what is observed in the single ΔRBCS mutant. Indeed, the expression of the reporter gene should depend only upon SSU in this hypothesis. Alternatively, the absence of SSU and LSU should lead to a high accumulation of the reporter in case of a negative feedback loop.

**Figure 4:**
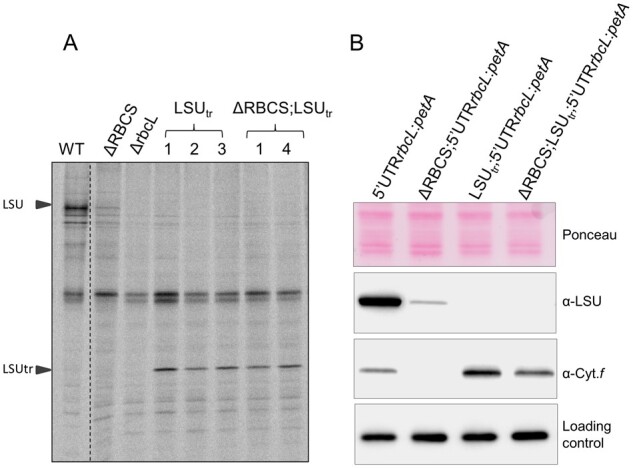
CES regulation does not occur in the absence of LSU accumulation. **(A)**^14^C labeling experiment showing synthesis rates of chloroplast proteins in WT, ΔrbcL, ΔRBCS, LSU_tr_ transformants (1-3), and ΔRBCS;LSU_tr_ (1 and 4) strains. Migration of full-length and truncated LSU is indicated on the left. The dashed line marks the position where two irrelevant lanes were removed. **(B)** Immunoblot depicting LSU and cyt *f* accumulation in representative transformants carrying both the 5′UTRrbcL:*petA* reporter gene and a truncation within the *rbcL* gene, associated or not to the ΔRBCS mutation, in comparison to the wild-type and ΔRBCS;5′UTRrbcL:*petA* strains. Ponceau stain and PsaD accumulation are shown as loading controls.

To test these two models, we first generated a strain in which LSU production was prevented. To this end, a construct bearing a deletion of 116* *aa was introduced at the *rbcL* locus, leading to the expression of a short, truncated polypeptide of 14* *kDa composed of the N-terminal part (107* *aa) fused in frame to the C-terminus (9* *aa). Biolistic transformation of the ΔrbcL (ΔR T1.3; mt+) and ΔRBCS (Cal.13.5A; mt+) strains by the pLStr plasmid carrying this truncation (“Materials and methods”; Supplemental Table S1) yielded strains wherein truncated LSU is expressed in presence or absence of SSU (LSU_tr_ and ΔRBCS;LSU_tr_). In vivo pulse labeling experiments revealed that truncated LSU is robustly synthesized in the LSU_tr_ transformants (Figure 4A). Furthermore, its synthesis rate was not altered in the absence of SSU (ΔRBCS;LSU_tr_). Yet, *rbcL* truncation led to the complete absence of LSU accumulation, which could not be detected even as trace amounts in the expected 14* *kDa size range (Supplemental Figure S2).

We next examined strains expressing truncated LSU in the reporter gene background with and without SSU (5′UTRrbcL:*petA* and ΔRBCS; 5′UTRrbcL:*petA*) (see “Materials and methods”; Supplemental Table S1). Figure 4B shows that the cyt *f* reporter becomes expressed to a significant level in the truncated LSU background (LSU_tr;_ 5′UTRrbcL:*petA* and ΔRBCS; LSU_tr;_ 5′UTRrbcL:*petA*) compared to the strains exhibiting native LSU (5′UTRrbcL:*petA)*. Its overexpression is observed irrespective of the presence of SSU (although not quite to the same extent in the ΔRBCS; LSU_tr;_ 5′UTRrbcL:*petA* as in the LSU_tr;_ 5′UTRrbcL:*petA* strain), in sharp contrast with the ΔRBCS; 5′UTRrbcL:*petA* strain in which cyt *f* did not accumulate (Figures 3, 4B). Thus the second model proved correct: unassembled LSU exerts a negative feedback on its own translation in Chlamydomonas, similar to what some of us had proposed for tobacco Rubisco (Wostrikoff and Stern, 2007).

### RAF1-LSU intermediates accumulate in absence of SSU

**Figure 5:**
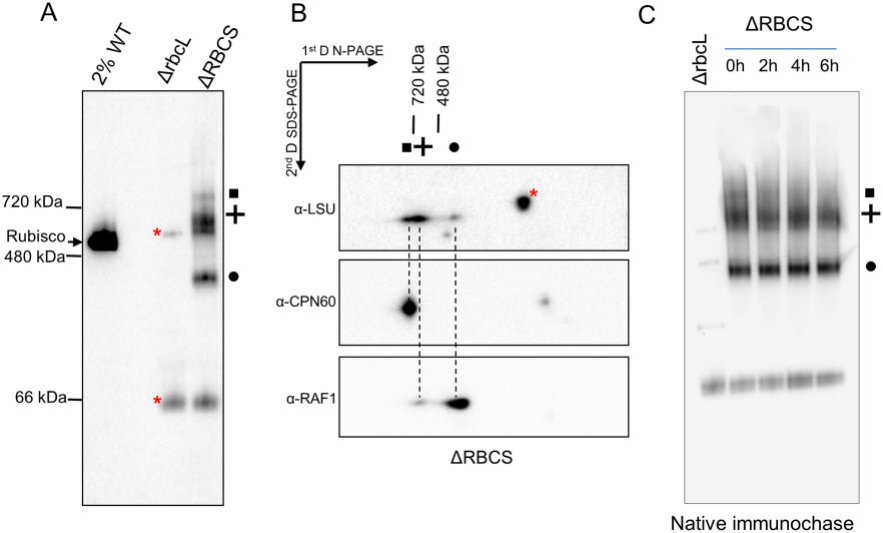
LSU assembly intermediates accumulate in the SSU-lacking strain. **(A)** Immunoblot with the antibody directed against Rubisco after native PAGE analysis of soluble protein extracts from WT (diluted to 2% as not to obscure the gel), ΔrbcL, and ΔRBCS strains. The migration of native molecular weight markers is indicated on the left. The position of Rubisco holoenzyme, as deduced from the WT signal, is indicated as well. Three LSU-specific complexes are observed in the SSU-lacking strain (depicted by a square, cross, and circle). (B) Analysis of the second dimension on SDS-PAGE gel by immunodetection of proteins putatively associated to LSU complexes in ΔRBCS strain (depicted by a square, cross, and circle as in (A), using anti-LSU, anti-CPN60, and anti-RAF1 antibodies. Dashed lines are drawn to help with the alignment. Red asterisks mark cross-contaminating signals of the anti-LSU antibody. (C) Immunochase in the ΔRBCS strain to follow the stability of the three LSU-oligomers detected in (A) (same symbols used) using a Rubisco antibody after native PAGE analysis as performed in (A). CAP, a chloroplast synthesis inhibitor, was added in the culture at the initial time point.

The Rubisco assembly pathway is suggested to comprise several LSU oligomerization steps followed by SSU binding (Liu et al., 2010; Hauser et al., 2015). To investigate in which oligomerization state the unassembled, repressor-competent form of LSU accumulates, we performed a native PAGE analysis of soluble extracts from whole cells. Immunoblotting against LSU readily detects the native Rubisco holoenzyme in a WT extract (2% dilution) (Figure 5A). No other assembly intermediates were detected even after prolonged membrane exposure, consistent with the idea that Rubisco assembly is a fast and dynamic process, which does not allow significant steady state accumulation of any assembly intermediate. Immunoblotting of ΔrbcL extracts revealed that the anti-Rubisco holoenzyme antibody cross-reacts with two LSU-unrelated bands, marked by asterisks on Figure 5A. These two bands are also found in the ΔRBCS extracts indicating further that they are not related to SSU. In the absence of SSU, the residual unassembled LSU (corresponding to about ∼1% of WT level, Figure 1) partitions into three distinct LSU-reactive complexes (Figure 5A, ΔRBCS lane). Using 2D electrophoresis and immunoblotting (Figure 5B), we identified a band migrating above 720* *kDa (depicted as a square in Figure 5A), which we attribute to the CPN60 chaperonin-bound LSU, as reported in previous studies with pea (Roy et al., 1982) and maize extracts (Feiz et al., 2012). A similar observation was reported regarding the CPN60 bacterial homolog GroEL in in vitro reconstitution experiments (Liu et al., 2010; Hauser et al., 2015). This attribution was confirmed with the use of a CPN60α/β1 directed antibody, which revealed two reactive bands. The upper one is found to co-migrate with the >720* *kDa LSU complex, whereas the lower one migrates in the molecular mass position of free CPN60 monomers. An immunochase in presence of chloramphenicol, a chloroplast translation inhibitor, revealed that the CPN60-LSU complex disappeared within 4* *h, whereas the other two LSU-associated complexes remained stable over 6* *h (Figure 5C). We then suspected that these two other LSU-associated complexes would be LSU oligomers bound to other assembly chaperones.

RBCX and RAF1 were shown to allow cyanobacterial LSU oligomerization in reconstitution experiments. While both RAF1 and RBCX are conserved in Chlamydomonas (Bracher et al., 2015; Hauser et al., 2015), we focused on RAF1 since it has been shown to interact in vivo with plant LSU (Feiz et al., 2012). We raised an antibody against Chlamydomonas RAF1 (Cre06.g308450, Supplemental Figure S3) and used it to detect a possible association with LSU complexes. After two dimensional electrophoresis of soluble ΔRBCS extracts and immunoblotting (Figure 5B), we were able to detect a RAF1 signal co-migrating with LSU in the two complexes below the 720 and 480* *kDa markers (depicted, respectively, by a circle and a cross). We noted that most of the signal is found in the lower molecular LSU complex (hereafter called LMW-LSU), whose apparent molecular mass would be consistent with an interaction between a RAF1 dimer and an LSU dimer.

**Figure 6:**
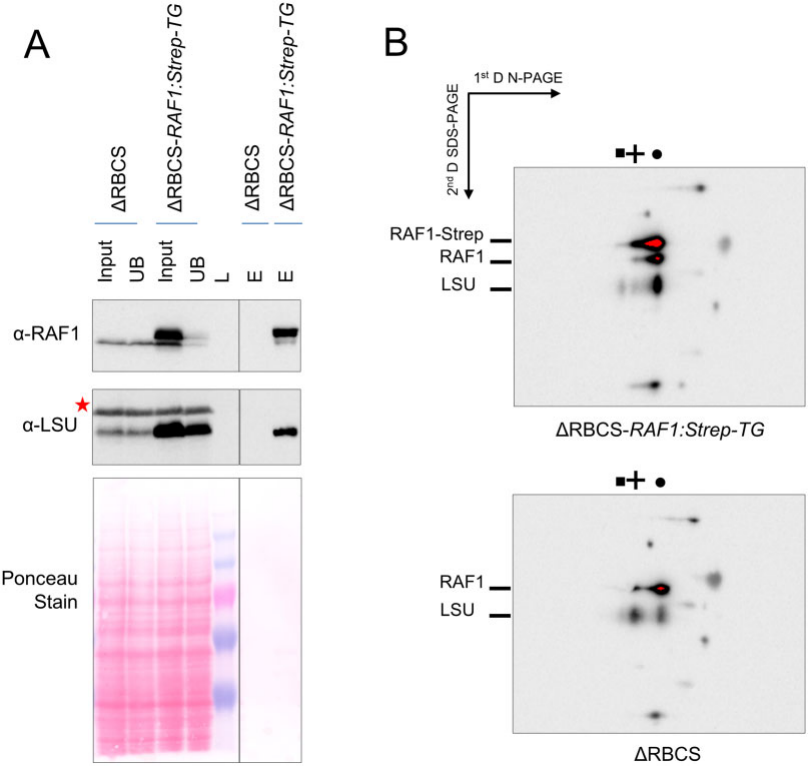
RAF1 and LSU interact in Chlamydomonas. (A) Immunoblots showing RAF1 and LSU co-immunoprecipitation. A similar fraction of the input, unbound (UB), or bead-extracted (E) fraction from the immunoprecipitation of soluble extracts from either the ΔRBCS or ΔRBCS-*RAF1:Strep-TG* strains was separated on SDS-PAGE gel, together with a molecular weight ladder (L). The line separates non-contiguous lanes of the same gel with the same exposure. RAF1 and LSU were detected by immunoblots using specific antibodies. The anti-RAF1 antibody recognizes both the endogenous (lower band) and the overexpressed epitope-tagged RAF1 (upper band), which could be separated by this gel system. The anti-Rubisco antibody recognizes LSU as well as an unrelated cross-reacting band marked by a red asterisk. LSU is specifically pulled-down by coimmunoprecipitation of the strep-tagged RAF1 protein. We note that not all LSU is pulled down, which could reveal the LSU fraction not associated to RAF1. **(**B**)** Strep-tagged RAF1 is associated to LSU LMW and HMW complexes, as shown by immunoblot using the RAF1 antibody and Rubisco antibody sequentially after separation of soluble proteins from the ΔRBCS-*RAF1:Strep-TG* and ΔRBCS on a 4–16% native gel followed by a second dimension in denaturating condition (10% SDS-PAGE, 8M urea gel). LSU complexes (depicted by a square, cross, and circle as in Figure 5A) in the ΔRBCS strain or ΔRBCS-*RAF1:Strep-TG* are shown on the top. Migration of the Strep-tagged or native RAF1 and of LSU is indicated on the left. The remaining observed signals come from cross-reactions with the antibodies. Note that here RAF1-related signals were left saturated, in order to properly see LSU-related signals.

To test whether the observed comigration of RAF1 and LSU is indeed due to a genuine interaction between the two proteins, we constructed an epitope-tagged version of RAF1 driven by a strong promoter (pJHL-RAF1S plasmid, see “Material and methods”) and transformed the ΔRBCS recipient strain. We recovered ΔRBCS; *RAF1:Strep-TG* transformants showing a three-fold overexpression of RAF1. An immunoprecipitation experiment from soluble extracts of one of these transformants and the ΔRBCS untagged control strain was then performed. After incubation with magnetic StrepTactin-coated agarose beads, the Strep-tagged RAF1 efficiently pulled down both tagged and untagged RAF1, as well as a significant part of LSU (Figure 6A). Thus we conclude that RAF1 and LSU are interacting in Chlamydomonas, as they do in land plants (Feiz et al., 2012). Moreover, the native and Strep-tagged RAF1 can oligomerize, either directly and/or through LSU. Further analysis of the soluble extracts isolated from the ΔRBCS; *RAF1:Strep-TG* strain and separated under native conditions (Figure 6B) revealed that the Strep-tagged RAF1 comigrates with the same LSU-containing complexes as those observed in Figure 5B. This confirms that co-immunoprecipitation of LSU and Strep-tagged RAF1 is not due merely to an artifact caused by RAF1 overexpression. Altogether these experiments show that (1) RAF1 genuinely interacts with LSU in Chlamydomonas, (2) this interaction is independent of the presence of SSU, and (3) it yields LSU-RAF1 complexes that accumulate in vivo, in agreement with reconstitution experiments performed with the recombinant cyanobacterial proteins (Hauser et al., 2015).

**Figure 7:**
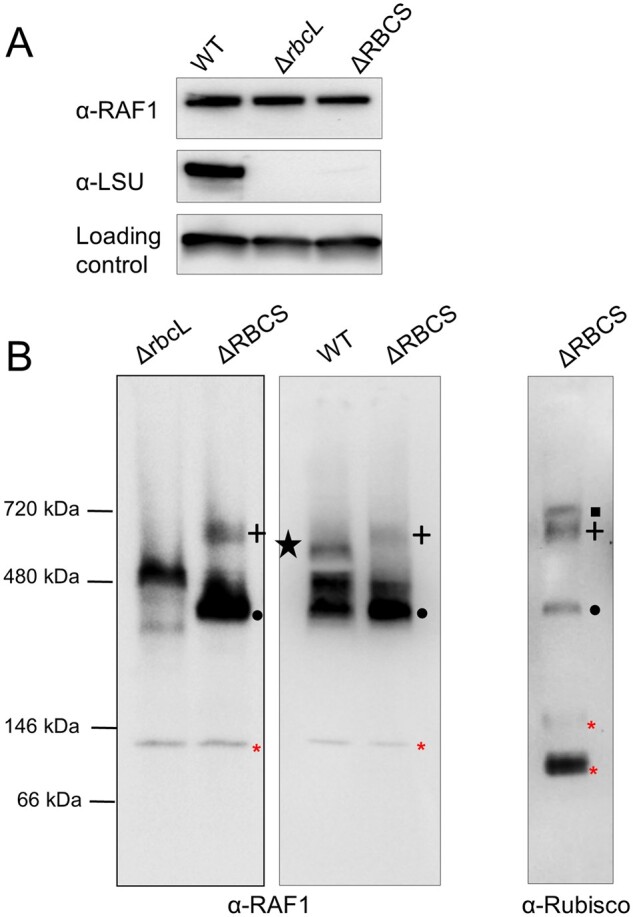
RAF1 oligomerization state in Rubisco mutants versus WT. (A) Immunoblot showing similar RAF1 content in *rbcL* or *RBCS* deletion mutants (ΔrbcL and ΔRBCS strains), and in WT, using antibodies directed against RAF1, Rubisco, and PsaD, as a loading control. Note that Rubisco accumulation was probed from a distinct membrane part obtained after the transfer of duplicated samples on the same gel. (B) Immunoblot of a 1D native PAGE of soluble extracts from ΔrbcL, ΔRBCS, and WT using RAF1 (left and middle panels) or Rubisco antibody (right panel), showing that RAF1 accumulates as an oligomer in the absence of LSU. RAF1-LSU complexes are indicated using the same symbols as in Figure 5. Note that the RAF1-LSU HMW complex found in the ΔRBCS is no longer detected in a WT background, whereas an additional low abundant RAF1 complex, indicated by a black star, is found. Red asterisks indicate antibody cross-reacting bands. (The left panel is a distinct experiment from the middle and right panels, which were separated on the same gel).

We next wondered whether these LSU-RAF1 complexes form only in the absence of SSU, or if they can be found in the WT as well, as expected from assembly intermediates. Consistent with the similar accumulation of RAF1 observed in the WT, ΔRBCS, or ΔrbcL strains (Figure 7A), both the HMW- and LMW-LSU associated RAF1 signals present in the ΔRBCS extract disappeared in the ΔrbcL extract, while a new RAF1 signal was detected above the LMW-LSU position (Figure 7B). Once more, these observations argue for a genuine interaction of RAF1 with LSU in these two bands, instead of a mere co-migration. Notably, we did not observe LSU-free RAF1 in the absence of SSU (Figures 5B, 7B), neither as a monomer nor as dimer, the latter being the form observed in solution when recombinant cyanobacterial RAF1 is produced (Hauser et al., 2015; Xia et al., 2020). These observations, together with the immunoprecipitation data, indicate that RAF1 plays a role in LSU stabilization in Chlamydomonas as has been reported for cyanobacterial LSU in in vitro studies. We suspect that RAF1 likely interacts with the LSU dimer, and part of it remains associated with higher LSU oligomers in vivo. Interestingly, while the LSU-RAF1 dimer also accumulates in a WT background, the LSU-RAF1 HMW observed in ΔRBCS is no longer detected. Instead another RAF1 HMW oligomer, showing a different migration pattern, is observed (as indicated by a star in Figure 7). How this might relate to the Rubisco assembly process and CES autoregulation will be discussed further below.

### CES regulation no longer occurs in Rubisco oligomerization mutants

**Figure 8:**
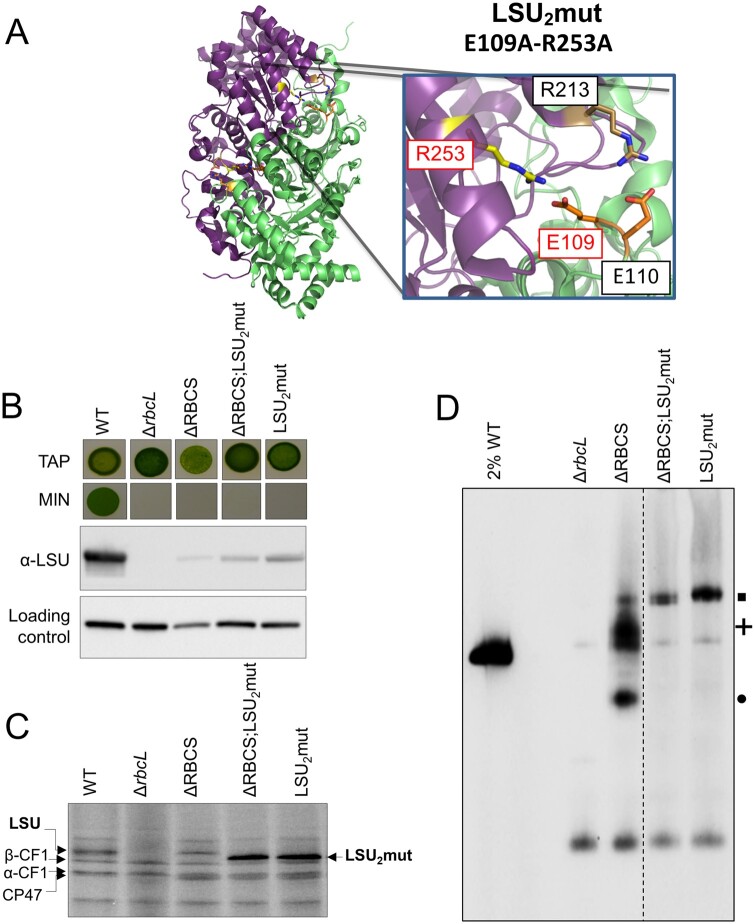
LSU_2_ mutations alter LSU accumulation and CES regulation. (A) Close-up of the mutated residues in LSU_2_mut strain in LSU structure. *C. reinhardtii* LSU dimer structure is shown in cartoon, as extracted from Rubisco structure (PDB: 1IR2). The two LSU subunits forming the dimer are represented in green and magenta. Subunits are maintained by two inter-subunits salt bridges between E109 and R253, and E110 and R213 residues. Residues mutated in LSU_2_mut (E109A and R253A) are highlighted in red. The figure was generated using the PyMol program (Schrödinger-LLC). (B) Impairment in Rubisco accumulation is revealed by the absence of phototrophic growth in the LSU_2_mut and ΔRBCS;LSU_2_mut strains as probed by spot tests on acetate-free minimal media (MIN). Growth on TAP is shown as a control. The corresponding soluble LSU accumulation detected by immunoblot is shown together with PsaD accumulation as loading control. (C) LSU synthesis rate in LSU_2_mut and ΔRBCS;LSU_2_mut measured by short ^14^C pulse labeling experiment and compared to WT. Note that in the 12–18% acrylamide-8M urea gel system, the mutated LSU undergoes a change in its migration pattern compared to native LSU. (D) Immunoblot with the Rubisco antibody after CN-PAGE analysis of soluble protein fractions of WT (note the dilution), ΔrbcL, ΔRBCS, LSU_2_mut and ΔRBCS;LSU_2_mut strains. A dashed line marks the position where two irrelevant lanes were removed. The position of the LSU-complexes observed in ΔRBCS is indicated at the right using the same symbols as in Figure 5 (square, cross, and circle).

To determine which LSU assembly intermediate is involved in the CES behavior of Rubisco, we undertook a structure-guided mutagenesis approach. LSU dimer interaction involves two stabilizing salt bridges between the E109 and R253 residues, and between the E110 and R213 residues from head-to-tail adjacent LSU monomers (Bracher et al., 2011) (see Figure 8A). To alter the formation of LSU dimers or their stabilization, we introduced two substitutions within the *rbcL* gene (E109A and R253A), aimed at preventing the formation of one of these salt bridges linking LSU monomers. The resulting LSU_2_mut (*rbcL*_E109A-R253A_) transformants were no longer phototrophic, as shown for a representative transformant in Figure 8B. One of these transformants (in mt+ background) was further crossed to ΔRBCS (mt−) to obtain a strain expressing these 2 *rbcL* substitutions in the absence of SSU. The resulting double LSU_2_mut-SSU mutant accumulates soluble LSU at comparable level to that in the single ΔRBCS mutant (Figure 8B). To monitor the rate of LSU synthesis in the dimerization mutants, we performed in vivo pulse-labeling experiment with ^14^C acetate as shown in Figure 8C for two representative progenies of the cross. In both LSU_2_mut and ΔRBCS;LSU_2_mut genotypes, the LSU translation rate was higher than WT levels, which is indicative of deregulated translation even in the complete absence of SSU. Further characterization by native PAGE and immunoblotting demonstrated that these mutations prevented accumulation of any LSU assembly intermediates beside the original LSU-CPN60 complex which in both genotypes is more abundant than in ΔRBCS (Figure 8D). No monomeric LSU could be detected, suggesting that dimerization is required to generate the chaperonin-independent, stable forms of LSU observed in Figure 5. We conclude that accumulation of some assembly intermediate, downstream of the CPN60-bound LSU complex is required for the CES regulation to occur.

**Figure 9:**
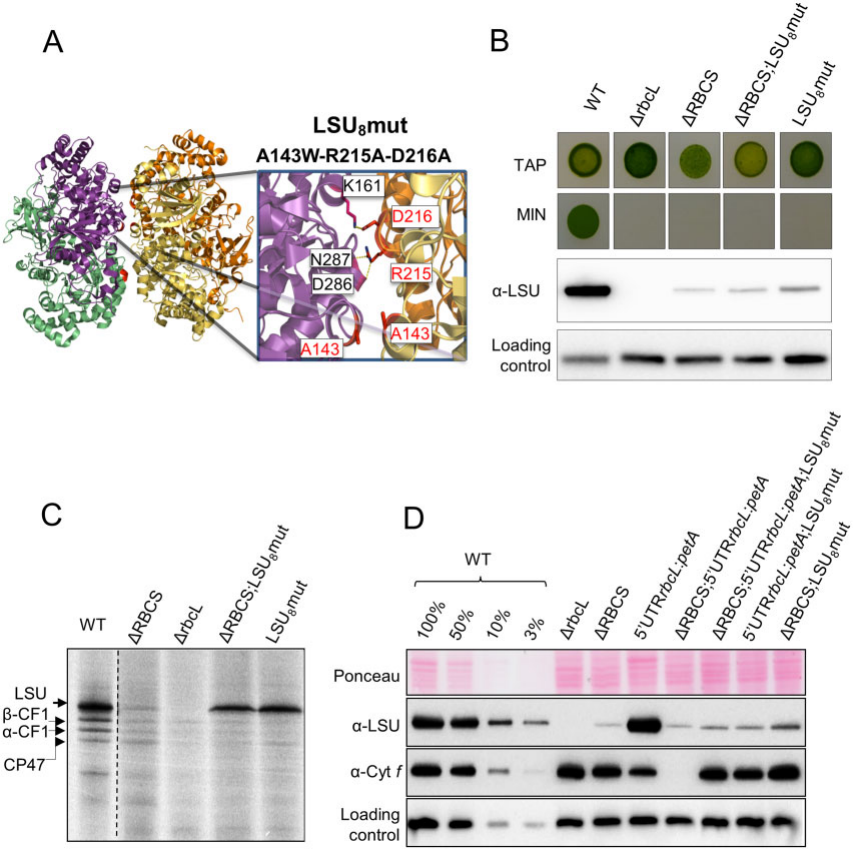
Disruption of LSU oligomerization alters LSU CES regulation. (A) Close-up of the mutated residues in the LSU_8_mut strain in LSU structure. Two LSU dimers facing each other are shown, as extracted from *C. reinhardtii* Rubisco structure (PDB: 1IR2). LSU subunits from the first and second depicted dimers are shown respectively in green and magenta, and in orange and yellow. The dimer to dimer interaction is stabilized by hydrogen bonds between the R215 and D286-N287 residues, and by a salt bridge involving the D216 and K161 residues, which are represented on the cartoon. The distance between the two dimers is the shortest at the A143 residues facing each other. Residues mutated in LSU_8_mut (ARD) are highlighted in red. The figure was generated using the PyMol program (Schrödinger-LLC). (B) Impairment in Rubisco accumulation is revealed by the absence of phototrophic growth in the LSU_8_mut and ΔRBCS; LSU_8_mut strains as probed by spot tests on acetate-free minimal media (MIN). Growth on TAP is provided as a control. Control strains WT, ΔrbcL, and ΔRBCS come from the same cultures as the ones used in Figure 7A. The corresponding soluble LSU accumulation detected by immunoblot is shown. **(C)** LSU synthesis rate in LSU_8_mut and ΔRBCS;LSU_8_mut measured by short 14C pulse labeling experiment and compared to ΔRBCS and WT. The dashed line marks the position of two irrelevant lanes, which were removed. (D) Immunoblot showing LSU and cyt *f* accumulation levels in the wild-type (WT), ΔRBCS, ΔrbcL, LSU_8_mut, and ΔRBCS;LSU_8_mut strains and in those latter three genetic contexts combined with the 5′UTRrbcL:*petA* reporter gene background. PsaD accumulation is provided as a loading control.

We then produced another set of mutations in the *rbc*L gene aimed at preserving the ability of LSU to dimerize but not to oligomerize further. According to the 3D structure of Rubisco (PDB 1IR2; Mizohata et al., 2002), the LSU octameric core shows stabilizing interactions between adjacent LSU dimers involving hydrogen bonds between the guanidino-group of R215 and carbonyls of the main chain of D286 and N287 residues, as well as a salt bridge between the D216 and K161 residues from adjacent LSU dimers. We further noticed that the distance between two LSU dimers was the shortest at the A143 residues which were facing each other closely at the interface of two LSU dimers. To prevent productive interactions between LSU dimers, we introduced a steric clash by replacing the A143 alanine with a bulky tryptophan (A143W, Figure 9A) and disrupted the hydrogen bond and salt bridge formation by replacing the arginine and aspartic acid charged residues by neutral ones (R215A-D216A, Figure 9A). The resulting triple A143W-R215A-D216A (ARD) substitution was introduced into the LSU sequence, yielding LSU_8_mut (rbcL_ARD_) transformants, which were produced both in an *RBCS +* (LSU_8_mut) or *RBCS* deficient context (ΔRBCS;LSU_8_mut). As expected and shown for representative strains from the different genotypes in Figure 9B, the LSU_8_ mutations resulted in a complete loss of phototrophy. Soluble LSU accumulated to levels comparable to that in a ΔRBCS strain, irrespective of the presence or absence of SSU (Figure 9B). We analyzed LSU translation rate in LSU_8_mut and ΔRBCS;LSU_8_mut by ^14^C pulse-radiolabeling (Figure 9C). In both cases, LSU was synthesized at a higher rate than WT, irrespective of the presence of SSU (compare LSU_8_mut lane and ΔRBCS;LSU_8_mut lane). Similarly to what had been observed for LSU_2_mut strains, the three substitutions abolished the CES behavior of LSU, allowing its translation to develop in an unregulated configuration.

To further substantiate this conclusion, we combined the same LSU_8_mut substitutions in the presence of the 5′UTRrbcL:*petA* reporter. To this end we introduced the ARD substitutions in a representative ΔRBCS;5′UTRrbcL:*petA* strain, which had undergone *aadA* marker removal (RCalΔK, see Supplemental Table S1). These transformants were crossed to the WT strain to segregate the Δ*RBCS* mutation and isolate progenies bearing the LSU_8_ mutations combined to the 5′UTRrbcL:*petA* reporter gene in presence or absence of SSU. We monitored translation of the *petA* reporter by analyzing cyt *f* accumulation. Figure 9D shows the results obtained for representative progenies, which should be compared to the original experiment shown in Figure 3, which demonstrated that the cyt *f* reporter showed a regular CES regulation when co-expressed with native LSU. In sharp contrast, when co-expressed with LSU bearing the ARD substitutions, the cyt *f* reporter now accumulated to the same extent, whether SSU was present or not (compare lanes “ΔRBCS; 5’UTRrbcL:*petA*;LSU_8_mut” and “5’UTRrbcL:*petA*;LSU_8_mut” to lane “ΔRBCS; 5’UTRrbcL:*petA*”). This shows that synthesis of the cyt *f* reporter is no longer regulated in the 5′UTRrbcL:*petA*;LSU_8_mut mutants, irrespective of the assembly state of Rubisco. Notably, cyt *f* accumulation accumulated to a higher extent when compared to the 5′UTRrbcL:*petA* strain producing native LSU. This indicates a higher translation rate of the reporter gene in the CES-insensitive context, and is similar to the increase of LSU synthesis rate observed in the LSU_8_ mutant alone compared to WT. Therefore, when Rubisco assembly can proceed, synthesis does not operate to its maximal rate, neither for LSU, nor for the cyt *f* reporter. Altogether, these observations suggest that there is still a significant translation repression in a WT context for LSU expression.

### Assembly intermediates in the LSU_8_mut oligomerization mutant reveal which LSU form is the CES repressor

**Figure 10:**
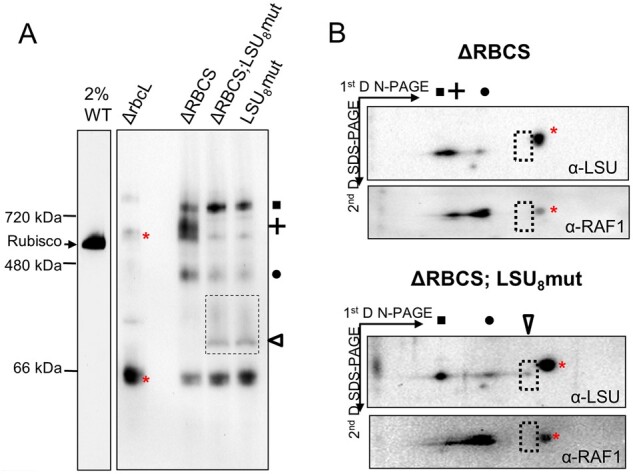
Alteration of CES regulation is concurrent with the disappearance of the LSU_8_-RAF1 oligomer. (A) Immunoblot with the Rubisco antibody after CN-PAGE analysis of soluble protein fractions of WT (note the dilution), ΔrbcL, ΔRBCS;LSU_8_mut, and ΔRBCS;LSU_8_mut strains. The position of the LSU-complexes observed in ΔRBCS is indicated at the right using the same symbols as in Figure 5 (square, cross, and circle). The empty triangle and dashed box indicate the somewhat diffuse band attributed to LSU dimer. (B) Immunoblot after CN-PAGE analysis (4–16%) of soluble protein fractions from ΔRBCS (top) and ΔRBCS;LSU_8_mut (bottom), followed by a second dimension run on a 13% SDS-PAGE gel using the anti-Rubisco and anti-RAF1 antibodies sequentially. The Rubisco antibody was stripped before rehybridization with the anti-RAF1 antibody, however a cross-reacting signal labeled with a red cross could not be completely stripped off. The position of the LSU-complexes observed in ΔRBCS and ΔRBCS;LSU_8_mut are indicated on top of the gels using the same symbols as in A (square: LSU-CPN60, cross: LSU_8_-RAF1 and circle: LSU_2_-RAF1). The empty triangle denotes the band observed in RBCS;LSU_8_mut attributed to RAF1-free LSU dimers. No corresponding signal can be detected at this position (dashed rectangle) with the RAF1 antibody in the ΔRBCS strain.

The LSU_8_mut mutants, with or without SSU, were analyzed by native PAGE in comparison with the ΔRBCS strain to characterize the pattern of accumulation of LSU intermediates (Figure 10A). Detection with the anti-Rubisco antibody yielded a different pattern for LSU_8_mut and ΔRBCS. The high molecular weight LSU-CPN60 complex observed above 720* *kDa and depicted as a square in Figure 10A was present and more abundant than in the ΔRBCS strain. The Rubisco-specific band that we attributed to LSU dimer bound to RAF1 (depicted as a circle) was still present, but slightly less abundant. However, a new Rubisco specific band of ∼100* *kDa, of low abundance and sometimes diffuse appearance, was observed, specifically in the two LSU_8_mut mutant strains (Figure 10A, open triangle). Due to its molecular mass, to the fact that it is not observed in the LSU_2_mut strains, and to the absence of its immunodetection with the RAF1 antibody (Figure 10B), this band most likely represents a chaperone-free LSU dimer rather than a monomeric LSU form bound to RAF1. Most interestingly, the high molecular weight LSU-RAF1 complex present in ΔRBCS (depicted as a cross in Figures 5, 10) was completely absent in the LSU_8_mut mutants. We conclude that the ARD mutations indeed prevented further oligomerization of LSU dimers into the LSU_8_ form, thereby preventing formation of the RAF1-containing HWM-LSU complex, which we tentatively attribute to an LSU_8_-RAF1 species. Thus, this HMW-LSU complex is likely to be the inhibitor of *rbcL* translation in ΔRBCS strain, as its specific disappearance caused by ARD mutations is concurrent with the escape from the CES regulation (Figure 9, C and D).

### The absence of MRL1 does not noticeably affect the migration of the LSU-HMW complex

We next wondered whether MRL1, the dedicated PPR protein involved in *rbcL* mRNA stabilization (Johnson et al., 2010; Johnson, 2011), could participate in CES regulation. MRL1 might behave as an effector of the translation inhibition by sequestering *rbcL* mRNA from the ribosome when SSU is not available for productive assembly. In this model, MRL1 would interact directly with the HMW-LSU complex. We therefore tested whether the migration of the HMW LSU-RAF1 complex was altered in absence of MRL1.

**Figure 11:**
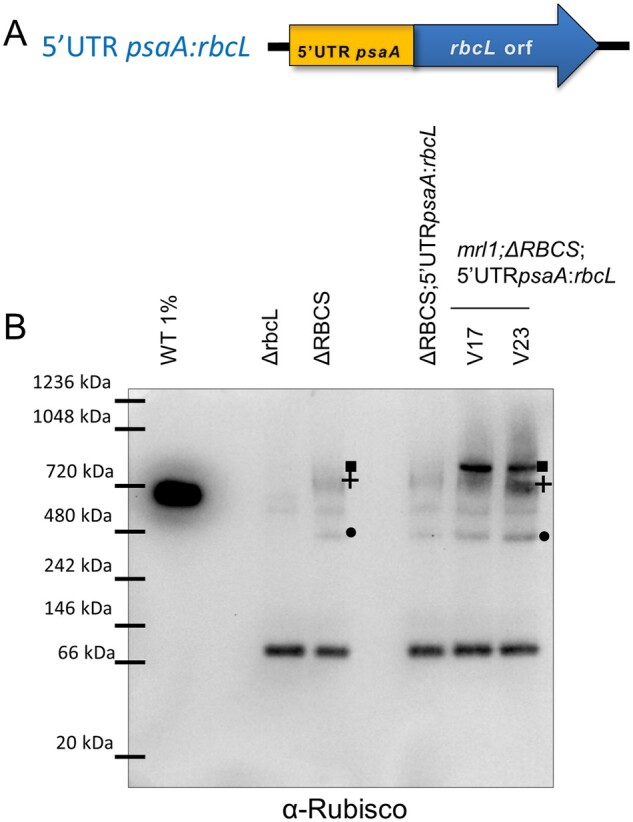
The LSU-HMW complex is not affected by MRL1 absence in the *mrl1*;ΔRBCS;5′UTRpsaA:*rbcL* strain. (A) Scheme of the 5′UTR psaA:*rbcL* chimeric construct. (B) Native immunoblot using the Rubisco antibody in order to follow the migration pattern of the LSU-HMW repressor complex using soluble proteins extracted from strains expressing LSU from the 5′UTRpsaA:*rbcL* chloroplast transgene in an *RBCS* mutant, in a *MRL1* WT background (ΔRBCS; 5′UTRpsaA:*rbcL*) or mutant background (*mrl1*; ΔRBCS; 5′UTRpsaA:*rbcL* V17 and V23). WT diluted extract, as well as extracts from the ΔrbcL and ΔRBCS strains, were included as controls. LSU oligomers are depicted by the same symbols used in Figure 5.

To produce LSU in an MRL1-independent manner, we used the 5′UTRpsaA:*rbcL* strain, and generated by successive crosses knock-out strains for *RBCS* and/or *MRL1* genes, placed in a 5′UTRpsaA:*rbcL* chloroplast context (*mrl1; ΔRBCS*; 5′UTRpsaA:*rbcL*). We next compared the migration pattern of the LSU-HMW repressor complex formed in the absence of SSU and in the presence or absence of MRL1. To this end, soluble proteins were extracted from strains expressing LSU from the 5′UTRpsaA:*rbcL* chloroplast chimeric gene in an *RBCS* mutant, either in a *MRL1* WT background (*ΔRBCS;* 5′UTRpsaA:*rbcL*) or in a *mrl1* mutated background (*mrl1; ΔRBCS*; 5′UTRpsaA:*rbcL* V17 and V23) (Figure 11). LSU oligomers were separated on a 1D-native gel and detected after immunoblotting with the Rubisco antibody in order to monitor a possible shift in the LSU-HMW complex in an *mrl1* mutated background (Figure 11B). Other than an increase in the CPN60-associated fraction in the absence of MRL1, no alteration of LSU oligomers’ migration—in particular the LSU-HMW complex depicted by a cross—was observed. This result suggests that MRL1 does not strongly interact with the HMW-LSU complex, yet we note that more labile interactions may have been lost in our experimental conditions. Therefore, the role of MRL1 in the regulatory process still awaits further characterization.

## Discussion

### New insights into the pathway for Rubisco biogenesis in vivo

**Figure 12:**
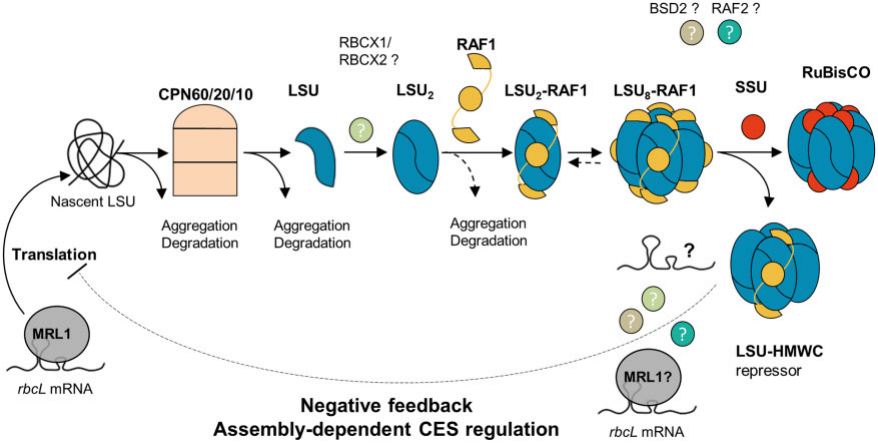
Model of Rubisco biogenesis pathway and CES regulation. The *rbcL* mRNA, stabilized by the binding of the MRL1 PPR-protein to its 5′UTR region, can be translated. Nascent LSU is recruited by the chloroplast folding machinery. LSU propeptide is subsequently folded in the CPN60/20/10 chaperonin complex. The released LSU dimerizes, maybe with help of RBCX, and recruits RAF1 which is required for LSU_2_ stabilization. LSU_2_-RAF1 unit oligomerizes further to form Rubisco catalytic core. RAF1 is finally substituted by the SSU to form the complete holoenzyme. In the SSU-limiting context, the LSU-RAF1 HMWC is converting to a repressor of *rbcL* translation (CES process) preventing LSU wasteful production, by binding either directly *rbcL* mRNA or other factors, thereby displacing some RAF1 oligomers. Many aspects of this model remain unclear such as the identity of the proteins/RNA in the LSU regulator complex, or the exact role of the other Rubisco assembly chaperones such as RBCX1/2 and RAF2, or the presence of a functional homolog of the plant BSD2 factor in algae, which remains debated.

Despite the simple final composition of the hexadecameric (LSU_8_SSU_8_) Rubisco enzyme present in cyanobacteria and chloroplasts of photosynthetic eukaryotes, at least five possible partners have been identified in its biogenesis pathway. Besides RBCX, all of these auxiliary proteins were identified in Rubisco-defective mutants (CPN60, (Barkan, 1993); BSD2, (Roth et al., 1996); RAF1, (Feiz et al., 2012), RAF2, (Feiz et al., 2014; Fristedt et al., 2018)), indicating their in vivo requirement for Rubisco biogenesis in the organism used for the genetic screen. In a few instances, biochemical assays pointed their interaction with LSU and/or SSU (Barraclough and Ellis, 1980; Feiz et al., 2012, 2014; Kolesinski et al., 2014). Here, we identified bona fide assembly intermediates in vivo using SSU-defective nuclear mutants and site-directed chloroplast mutants of LSU in Chlamydomonas. Our analysis of a ΔRBCS strain allowed us to detect low amounts of LSU assembly intermediates that would otherwise be obscured by the hundred times more abundant fully assembled holoenzyme. This revealed the existence of two LSU-containing species, besides the CPN60-LSU complex previously identified in plants either by in organello translation (Roy et al., 1982) or in maize chloroplasts (Feiz et al., 2012). As summarized in Figure 12, our work supports a pathway whereby newly-synthesized LSU, arising from the translation of an MRL1-protected *rbcL* mRNA, must be kept unfolded, maybe with the help of general chaperones such as a trigger factor (Rohr et al., 2019), until its loading on the CPN60 chaperonin. Its release would be followed by a rapid dimerization, possibly assisted by the RBCX chaperone. RAF1 would subsequently bind, leading to the stabilization of an LSU_2_-RAF1 intermediate. Subsequently, oligomerization would proceed up to an LSU_8_ core, still RAF1-associated, before SSU binding in the ultimate step of Rubisco assembly, thereby displacing the bound chaperone. That SSU requires an LSU octamer for binding is readily deduced from our observations of (1) a similar accumulation of LSU intermediates, whether SSU is present or not, in the LSU_8_ oligomerization mutant and (2) a similar pattern of LSU intermediates in the LSU_2_ mutant, irrespective of SSU availability.

This biogenesis pathway, which results from the present in vivo work on the Chlamydomonas enzyme, is further supported by several in vitro and in vivo studies of the cyanobacterial and plant enzymes (Saschenbrecker et al., 2007; Liu et al., 2010; Bracher et al., 2011; Hauser et al., 2015; Aigner et al., 2017). The proposal that RAF1 interacts with LSU in Chlamydomonas (Figure 6) is consistent with its interaction with LSU in cross-linked maize extracts (Feiz et al., 2012) and in cyanobacteria (Kolesinski et al., 2014; 2017). In agreement with in vitro studies of cyanobacterial LSU (Kolesinski et al., 2014; Hauser et al., 2015), we provided several lines of evidence that the RAF1_2_-LSU_2_ dimer, close to 200* *kDa (2* *×* *52* *kDa LSU* *+* *2* *×* *51* *kDa RAF1* *=* *206* *kDa), represents indeed a genuine intermediate in the pathway for Rubisco assembly: (1) it was easily detected in a mutant lacking SSU but it was also detected in WT extracts (Figure 7B), and (2) it was absent from an LSU dimerization mutant (Figure 8D) but it accumulated in absence of further LSU oligomerization, as in the LSU_8_ mutant (Figure 10A). We note however that LSU dimers without RAF1 still can be formed (Figure 10, A and B). This suggests that RAF1 is not required for the formation of LSU dimers per se but rather for their stabilization.

Interestingly, in organello pulse-labeling experiments in pea chloroplasts—which have limited availability of unassembled SSU or chaperones—also identified a 7S LSU-associated complex attributed to an LSU dimer (Hubbs and Roy, 1992) and an LSU_8_-like species called Z (Hubbs and Roy, 1993). The size of these two complexes is consistent with that of the LSU oligomers that we identified in this study (Hubbs and Roy, 1993). Notably, we and others (Hubbs and Roy, 1992, 1993; Feiz et al., 2012) never detected LSU monomers. These were also absent in the LSU_2_ dimerization mutant despite the enhanced synthesis of LSU in pulse-labeling experiments (Figure 8). Therefore, LSU monomers are either not proteolytic-resistant or not sufficiently soluble to accumulate to detectable levels.

The LSU_8_-RAF1 complex, migrating above the holoenzyme with an apparent molecular mass of ∽720* *kDa, is present in ΔRBCS, but not in the LSU_8_ oligomerization mutant (Figure 10A). The size of this complex is close to the observed size of the LSU_8_-RAF1_8_ complex identified in reconstitution experiments performed in vitro in the absence of SSU, using denatured cyanobacterial LSU from *S. elongatus* sp. PCC7942 and RAF1 (Hauser et al., 2015). This LSU_8_-RAF1_8_ complex is believed to constitute the end-point assembly intermediate prior to SSU binding in cyanobacteria. Other studies (Kolesinski et al., 2014; 2017; Xia et al., 2020) also have suggested a role for RAF1 in the dissociation of assembled Rubisco in cyanobacteria, where the RAF1-LSU_8_ complex would be a breakdown product of Rubisco holoenzyme. However, we observed that this form is long-lived in an SSU-lacking strain (Figure 5C), which better fits a model where the RAF1-LSU_8_ complex is an assembly intermediate rather a degradation product of LSU-octamers. Here, we observed by immunoblotting a higher LSU/RAF1 labeling ratio in the 720* *kDa than in the 200* *kDa oligomers (Figure 5). This result suggests that there may be as yet unknown interactants in the LSU_8_-RAF1 complex, unless RAF1 is more easily lost from the LSU_8_ core than from LSU_2_ during their purification. The actual composition of the LSU_8_ oligomers before binding of SSU remains confusing. LSU_8_ has been crystallized with RBCX (Bracher et al., 2011), RAF1 (Huang et al., 2020; Xia et al., 2020), and BSD2 (Aigner et al., 2017). However, the last assembly intermediate is considered as a RAF1_8_-LSU_8_ complex in cyanobacteria (Hauser et al., 2015; Kolesinski et al., 2017; Xia et al., 2020), but as a BSD2_8_-LSU_8_ complex in plants (Aigner et al., 2017; Conlan et al., 2019). In view of our results and of the absence of a dedicated BSD2 factor in green algae, we posit that the RAF1-LSU_8_ complex constitutes the penultimate assembly intermediate in green algae. Interestingly, the RAF1-LSU_8_ complex in Chlamydomonas, when probed with the antibody against RAF1, exhibits a different migration pattern depending on SSU availability (Figure 7B). This raises the possibility of some flexibility in the composition of RAF1-LSU_8_ oligomers depending on the availability for SSU.

### Fate of unassembled LSU

In the absence of SSU, which prevents Rubisco assembly, LSU synthesis is inhibited but not fully impaired, as newly-synthesized LSU is readily detected in pulse-labeling experiments (Figure 1). Yet the LSU accumulation level drops to <1%, as estimated by comparing the LSU signal in ΔRBCS to a WT dilution series. We observed that unassembled LSU is stable over >4* *h, as shown by immunochase experiments (Figure 1D;Supplemental Figure S1). Our study showed that the discrepancy between reduced LSU synthesis and a much larger drop in its accumulation in the absence of SSU results from both an inhibition of synthesis dependent of the 5′UTR and from the degradation of excess unassembled LSU, independently of the 5′UTR. This is further exemplified by the observation that even with a high rate of synthesis for native LSU, as observed in the ΔRBCS;5′UTRpsaA:*rbcL* strain, the maximal amount of unassembled LSU, accumulating as soluble protein, reaches <10% of the WT level. Altogether, it suggests a bottleneck in LSU assembly, likely due to the limiting amount in one of the assembly chaperones: part of neo-synthesized LSU undergoes rapid proteolysis, when not stabilized by assembly factors. Our data suggest that RAF1 is in limiting amounts. Indeed, we noted that a fraction of LSU dimers is not found associated with RAF1 in the LSU_8_ oligomerization mutant, where LSU is produced in excess (Figure 10, A and B). That the LSU mutations would rather alter the affinity constant for RAF1 binding, thereby displacing the equilibrium between the RAF-bound and unbound forms of LSU dimers, is unlikely since the mutated residues do not belong to the RAF1-LSU interface regions identified in the recently obtained LSU_8_-RAF1_8_ complex crystals (Xia et al., 2020). Rather, this suggests that RAF1 is present in limiting amounts, thereby leading to accumulation of LSU dimers without the chaperone. In support of this conclusion, we observed a dramatic increase in the accumulation of unassembled LSU concomitant to RAF1 overexpression using RAF1-epitope-tagged strains (compare input fractions of tagged strains versus WT in Figure 6). Similarly, RAF1 overexpression in maize lines led to a 30% increase in whole Rubisco content (Salesse-Smith et al., 2018), albeit understandably only when SSU is overexpressed as well.

We further note that the LSU_8_ oligomerization mutant and LSU_2_ dimerization mutant display a similar drop in the accumulation of LSU as in ΔRBCS despite their much higher rates of LSU synthesis. Thus the proteolytic susceptibility of LSU is increased in these mutants (Supplemental Figure S1). One should consider also a possible formation of LSU-insoluble aggregates that would escape recovery in our gels used for electrophoretic purification. Such aggregates have been described by (Knopf and Shapira, 2005) under oxidative stress, or by Zhan et al. (2015) in the ΔRBCS strain. Although we did not detect such aggregates in ΔRBCS, we observed some triton-insoluble LSU in the two oligomerization mutants, as well as in the 5′UTRpsaA:*rbcL* expressing strain (data not shown). Whether these result from LSU unproductive interactions prior to LSU loading on the CPN60/CPN23/CPN10 complex, or after its release from the chaperonin is still unknown. We observed in those strains, along with a higher translation rate of the *rbcL* transcript, an increase in the abundance of the CPN60-LSU complex when compared to ΔRBCS (Figure 10A), suggesting that the chaperonin is not in limiting concentration. Altogether, these observations indicate that there may be several quality checkpoints in Rubisco folding before and after chaperonin interaction, thus directing excess, chaperone-free unassembled LSU subunits either to degradation and/or to aggregation, as depicted in Figure 12.

### The CES process for LSU

Efficient biogenesis of the oligomeric proteins which build up the photosynthesis apparatus requires coordination in the expression of their subunits; even more so when their subunits are encoded in distinct intracellular compartments, precluding transcriptional co-regulation as is the case in photosynthetic bacteria. Indeed, we previously demonstrated that a number of chloroplast genes encoding core subunits from photosynthetic complexes in Chlamydomonas undergo regulatory loops depending on their assembly state with the other subunits of the same protein complex. This feedback, called the CES process (Wollman et al., 1999; Choquet and Wollman, 2007), occurs at the level of translation initiation. Its importance is reflected by its prevalence, as CES subunits have been identified in all membrane-embedded photosynthetic proteins, PSI and II, cyt *b_6_f* and ATP synthase, allowing fine-tuning of their expression by the presence of their assembly partners. Here, we showed that Rubisco also displays CES behavior for its biogenesis in Chlamydomonas: LSU synthesis is consistently reduced in absence of SSU, although we noticed some variability between experiments (Figures 1B, 2B, 4A, 8C, 9C), which likely reflect differences in physiological state of the strains and changes in the rate of cellular uptake of radiolabeled carbon. Rubisco is a most interesting case, as CES behavior for LSU also has been observed in higher plant chloroplasts (Wostrikoff and Stern, 2007), providing an example of the conservation of this regulatory process in multicellular eukaryotes. Chlamydomonas offers unique opportunities to shed more light on the CES mechanism for Rubisco because it is amenable to genetic approaches. This allowed us to perform experiments with Chlamydomonas that presently are not feasible in plants, to provide a complete demonstration of the control of *rbcL* expression by the state of LSU assembly.

We first showed that the CES process for LSU synthesis is exerted at the level of initiation of translation. We came to this conclusion after swapping *rbcL* 5′UTR. We demonstrated that this gene region contains all cis-elements required for the assembly-dependent regulation of *rbcL* translation. This excludes both an effect on translation elongation and an early co-translational degradation of LSU, both of which would target its coding sequence and not the untranslated region of the gene. This is a feature which distinguishes the CES process from the other known translational regulation of LSU, which were attributed to a change in its rate of translational elongation such as LSU inhibition of translation elongation in the dark (Mühlbauer and Eichacker, 1998) and LSU translational repression under oxidative stress (Shapira et al., 1997). In the latter case, a structural conformational modification in oxidized LSU was suggested to expose an otherwise buried N-terminal domain. This domain adopts a ferredoxin fold structure, similar to an RNA binding domain, and was found to have an unspecific RNA binding capacity (Yosef et al., 2004; Cohen et al., 2005).

As the CES process results from the autoregulation of translation by LSU, it should be mediated by either one of the three stable assembly intermediates found to accumulate in absence of SSU. The first step of LSU folding, CPN60 bound LSU, does not serve as a regulator of translation since the two oligomerization mutants that we tested accumulated higher level of this intermediate but showed no translational repression despite the absence of Rubisco assembly. By contrast, the LSU_8_-RAF1 complex displays the properties expected for a negative-feedback regulator: its absence correlates with the escape from the CES process in both the dimerization and LSU_8_mut mutants (*rbcL*_E109A-R253A_ and *rbcL*_ARD_). Interestingly, our data also ruled out the possibility that the repressor would be constituted by free monomeric LSU or by the LSU dimer which is not compromised in the LSU_8_mut mutant. Thus, the LSU octamer accumulated in absence of the SSU partner is the key LSU form which provides the assembly-dependent translational control.

Whether RAF1 plays an active role in the regulation of *rbcL* translation, or only mediates the formation of the HMW-LSU repressor form remains to be determined. Notably, in maize, where LSU is also under CES regulation (Wostrikoff et al., 2012), the RAF1 knock-out mutant displays a high LSU synthesis rate (Feiz et al., 2012). This observation is compatible with RAF1 being required for the formation of the translational repressor, but does not reveal RAF1’s role in the repression. Facts detracting RAF1 direct involvement in this process come from its unexpected stoichiometry to LSU. While we could not retrieve from our data the exact stoichiometry of RAF1 to LSU in the repressor form, it seems unlikely that the repressor complex comprises 8 LSU subunits bound to 4 RAF1 dimers, as the RAF1 to LSU ratio is decreased in the LSU_8_-RAF1 complex compared to the LSU_2_-RAF1 dimer. This is at variance with cyanobacterial reconstitution experiments that identify the LSU_8_-RAF1_8_ complex to be stable (Hauser et al., 2015). As noted above, there is a slight but significant difference in migration of the HMW-RAF1 complex in the WT compared to the ΔSSU strain (Figure 7). Additional experiments on this issue are challenged by the presence of large amounts of Rubisco complexes overlapping with LSU_8_-RAF1 in the WT. It is tempting to consider that it corresponds to the LSU_8_-RAF1_8_ assembly intermediate, which has been observed and crystallized in cyanobacteria (Hauser et al., 2015; Xia et al., 2020), which would convert to a repressor form complex in absence of SSU, leading to this slight change in migration in our gel system. The observed upshift may result from the capture of other proteins or RNA thereby leading to translational repression (Figure 12).

The molecular mechanism by which LSU_8_-RAF1 controls translation of the *rbcL* transcript could result from the RNA binding activity of Rubisco RNA Recognition Motif that would allow the *rbcL* mRNA to get sequestered in this complex, thereby rendering it unavailable for translation. In such a case, repression would result from a direct interaction between LSU and its transcript. Whether the RRM domain is indeed accessible in the repressor conformation cannot be reasonably addressed without better knowledge of the components of this repressor form, which is a challenge owing to its very low accumulation level. Alternatively, CES translation inhibition could be mediated by an additional trans-acting factor, as has been documented for the CES process governing cyt *f* translation. In this model, the MCA1 protein that is responsible for *petA* mRNA stabilization and translation is targeted to degradation via its interaction with a repressor motif exposed when cyt *f* remains unassembled (Boulouis et al., 2011). The MRL1 factor is an obvious candidate for this function: it interacts with *rbcL* mRNA and promotes its stabilization. Furthermore, MRL1 is found in a large complex, whose size is dependent upon LSU presence (Johnson et al., 2010). Yet, the proposed mechanism would be different as we found MRL1 to be stable at variance with MCA1 (data not shown). MRL1 could be part of the LSU repressor complex, thereby leading to *rbcL* transcript sequestration away from the ribosomes. However, in this model, the absence of MRL1 should alter the migration pattern of the LSU_8_-RAF1 complex on native gels, which is not observed (Figure 11). Alternatively, an as for now undetected interactant, such as RBCX, could indirectly mediate this interaction. Interestingly, a similar model has recently been proposed to be involved in D1 translation in higher plant chloroplasts. The presence of a D1-HCF244-OHP1/2 assembly intermediate was linked to the inhibition of D1 synthesis in the dark, relieved in the photorepair process. This assembly intermediate has been suggested to act as a repressor complex which may physically interact with D1 HCF73 translational activator to mediate D1 repression in the dark (Chotewutmontri and Barkan, 2020).

In conclusion, our suggestion that the repressor form identified in the absence of assembly constitutes a regulatory pool, rather than representing a true end-state assembly intermediate in Chlamydomonas, further raises the question of whether it would participate in regulating LSU synthesis rates in regular WT conditions. The large amount of assembled Rubisco precludes us from testing whether the same LSU_8_-RAF1 complex is present in WT. However, the observation that the LSU synthesis rate may be higher than what is observed in WT conditions, as observed in the dimerization or oligomerization mutants, hints to the fact that LSU synthesis is probably limited by a CES mechanism even in WT, as has been reported for cyt *f* (Choquet et al., 1998; Choquet and Wollman, 2007). Further experiments to determine the exact composition of the LSU_8_-RAF1 complex are required in order to discover the precise mechanism of assembly-mediated *rbcL* translation inhibition and confirm whether or not it occurs in WT conditions.

## Materials and methods

### Cultures and strains

If not stated otherwise, WT (WT.T222 mt+ and WT.S24 mt−, (derived from 137c: nit1 nit2) and mutant strains of *C. reinhardtii* were grown on solid Tris-acetate-phosphate medium (TAP, pH7.2) (Harris, 2009) supplemented with agar and in liquid cultures under continuous, dim light (7* *µmol photons.m^−2^.s^−^1, white light-emitting diode, whose emission spectrum is shown in Supplemental Figure S4) on an orbital shaker (120* *rpm) at 25°C. Cells from exponentially growing cultures (2* *×* *10^6^ cells.mL^−1^) were used for all experiments.

### Chlamydomonas genetics

Chlamydomonas mating and progeny isolation were done as by Harris (2009). The *ΔRBCS;* 5′UTRpsaA:*rbcL* strain is a progeny from the cross 5′UTRpsaA:*rbcL*, mt+ x *ΔRBCS*-Cal13.1B, mt−; while the *mrl1; ΔRBCS*; 5′UTR psaA:*rbcL* strains were obtained in the cross *mrl1;* 5′UTRpsaA:*rbcL*, mt+ x *ΔRBCS*-Cal13.1B, mt−. All strains were probed by PCR to ascertain their correct genotype.

### Nucleic acids manipulations

If not stated otherwise DNA manipulations were done following standard protocols as by Sambrook et al. (1989). RNA extractions and blotting was performed as by Drapier et al. (2002).

### Plasmids and strains preparation

Plasmid pRFFFiK aimed to express the *petA* gene from *rbcL* 5′ regulatory regions was described by Johnson et al. (2010). Plasmids carrying mutations aimed to introduce a truncation (pLStr) or a triple ARD substitution in LSU sequence (pLS_ARD_), to prevent LSU dimerization (pL_2_mut), or carrying the *psaA*-driven *rbcL* gene (paAR) described below all contain the *5′psaA-aadA-atpB* 3*′* selection marker conferring resistance to spectinomycin, flanked by direct repeats (Fischer et al., 1996) allowing the cassette removal in absence of selection pressure, at neutral positions either at *rbcL* 5′(*Bse*RI site) or 3′end (*Afl*II site). To generate this *aadA* excisable cassette, the paAX plasmid described by Wostrikoff et al. (2004) was modified to replace the *rbcL* 3′ regulatory region with the one of *atpB*. To this end, *atpB* 3′UTR was PCR-amplified and flanked by *Pst*I and *Spe*I restriction sites using the atpB Pst.F and atpB Spe.R primers, and cloned into *Pst*I-*Spe*I digested paAX plasmid, yielding the paAEXCdB plasmid.

pLS_ARD_ plasmid was generated using the In-Fusion PCR Cloning Kit (Clontech, In-Fusion^®^ HD Cloning Plus), following the manufacturer’s instructions, from the R15 plasmid backbone (Johnson et al., 2010) amplified with the IP-R15 lin.F and R primers, and a 252* *bp region from R15 amplified with primers IP-LS-A143.F and IP-LS-R215D216.R introducing the A143W, and R215A and D216A mutations, respectively. The *aadA* excisable cassette from paAEXCdB plasmid was further subcloned by *Kpn*I and *Sac*I digestion, followed by blunting using the NEB Quickblunting kit and ligation into *Afl*II-digested pLS_ARD_ plasmid, or R15 plasmid. Clones in which the *aadA* cassette inserted in a reverse orientation compared to the *rbcL* gene were selected, yielding pLS_ARD_-X and pR15-X3′ respectively.

pL_2_mut plasmid was similarly assembled from an R15 PCR-amplified fragment using the IP-R15 E109.R and IP-R15R253 *Pst*.F primers, and a 473* *pb amplified fragment containing the mutated *rbcL* region containing the E109A and R253A substitutions introduced with the IP-LS E109A Bam.F and IP-LS R253A P.R primers. The *aadA* marker (*Kpn*I-*Sac*I fragment of paAXdB, blunted) was thereafter introduced at the *Bse*RI site upstream of the *rbcL* promoter in reverse orientation compared to *rbcL*, yielding the pLS_2_mut-X plasmid. To check that the cassette insertion is neutral on *rbcL* expression, the aAEXCdB marker was also introduced in the pR15 plasmid, yielding pR15-X5′.

The pLStr plasmid was generated from the pLS_ARD_-X plasmid by *BstB*I digest followed by subsequent religation.

To generate the paAR plasmid, the *psaA* promoter region was first fused to part of *rbcL* CDS sequence by overlapping PCR using the following primer pairs: PsaAProm.F/psaAProm-rbcL.R, and psaAPromRbcL.F/RbcL EcoNI.R., respectively, on the paAEXCdB and R15 plasmid templates with the Phusion Taq polymerase (NEB). The resulting 814* *bp fragment was further amplified using the IP-psaAProm.F and IP-rbcL EcoNI primers, and assembled into the R15 backbone amplified by the IP-R15 BseRI.R and IP-R15 EcoNI.F2 primers using the Clontech In-Fusion PCR Cloning kit. Insertion of the aAEXCdB excisable marker (*Kpn*I-*Sac*I blunted fragment) was further performed at the *BseR*I restriction site, yielding paAR-X plasmid in which the *aadA* marker is in opposite orientation compared to *rbcL*.

All clones were sequenced and no mutations were found within the chloroplast containing sequences. All primer sequences are displayed in Supplemental Table S2.

### Generation of chloroplast transformants

Chloroplast transformation was done as described by Kuras and Wollman (1994) using an in house built helium-driven particle gun. Recipient strains, specified in Supplemental Table S1 are: the WT T222 mt+ strain, the *rbcL* deletion strain ΔR T1.3 mt+, the *RBCS* mutant Cal.13.5A mt+ strain (back-crossed progeny of the CAL005.01.13 strain described by Dent et al. (2005)), and RCalΔK strain (Cal13.5A mt+ transformed with pRFFFiK [containing the *petA* sequence where the endogenous *petA* 5′UTR was swapped by the *rbcL* promoter and 5′UTR, described by Johnson et al. (2010)] and subjected to cassette removal (Fischer et al., 1996)). Transformants were brought to homoplasmy by six rounds of subcloning on selective media (TAP supplemented with 500* *μg/mL Spectinomycin), after which homoplasmy was confirmed by PCR analysis. Primers used to check the state of homoplasmy of the aAR transformnats (PsaAprom.F/dRLS.R1 and dRLS.F/dRLS.R2), of the LS_ARD_ transformants (LSmutA143W.F/LSmutD216A.R and LSA143wt.F/LSD216wt.R), of the L_2_mut transformants (LSmutE109A.F/LSmutR253A.R and LSE109wt.F/LSR253wt.R), and of the LStr transformants (CrRbcL Prom.F2/CrRbcL.R3 and CrRbcL Prom.F2/CrRbcL EcoNI.R) can be found in Supplementary Table S2. As a rule, three independent transformants were further analyzed and showed negligible phenotypic variation.

### Generation of Strep-tagged RAF1 transformants

Cloning of the pJHL-Raf1S plasmid, allowing expression of the RAF1 gene fused at its C-terminus to a StrepII tag driven by the PsaD promoter and carrying the *aphVIII* resistance gene was performed as follows. *RAF1* (Cre06.g308450) predicted gene model from Chlamydomonas genome version 5.6 was first curated and extended to an in-frame Met at the Nter found 147 nt upstream, yielding a protein with 49 additional amino-acids, predicted to be chloroplast targeted by Predalgo (Supplemental Figure S3A) (Tardif et al., 2012). The curated coding sequence was synthesized omitting its first four introns and keeping only the last intron, and fused to an 18 aa long Gly-rich linker (S-G_4_-S-G_4_-S-M-G_4_-S-N) followed by a StrepII tag at the C-terminus (WSHPQFEK). *Eco*RI and *Bam*HI restriction sites were, respectively, included at the 5′ and 3′extremities of the RAF1 coding sequence, which was cloned in the pBS-SK backbone yielding CrRAF1S-pBSII (GeneCust, France). The *Eco*RI-*Bam*HI RAF1-Strep fragment was then subcloned in the pPEARL backbone, digested by the same enzymes, thereby placing *RAF1* coding sequence under the control of the *RBCS2* promoter, *RPL17* 5′untranslated region and *PsaD* 3’ untranslated region. The pPEARL backbone also contains the *aphVIII* resistance gene expressed from the combined *RBCS2* and *PsaD* promoters and the *RBCS2* terminator region (Takahashi et al., 2016). Nuclear transformation of the ΔRBCS mutant by the pJHL-RAF1S plasmid was performed according to Onishi and Pringle (2016), and transformants selected on TAP supplemented with 10* *μg/L paromomycin. RAF1 expressing transformants were screened for integration of the RAF1 coding sequence by PCR using the primers CrRAF1.F2 and PsaD.R. Three independent transformants displaying strong RAF1 expression as evidenced by immunoblot analysis using the RAF1 antibody (1/60.000 dilution) were selected.

### Pulse experiment

Chlamydomonas ^14^C-acetate pulse experiment was done as described by Drapier et al. (2007). A total of 5* *×* *10^6^ cells were washed once in MIN-Tris medium then resuspended in 5* *mL fresh MIN–Tris medium and incubated for 1* *h at RT with vigorous shaking to remove the acetate from the medium at a light intensity of 30* *µmol photons.m^−2^.s^−1^. Subsequently, 5* *µL of 1* *mg.mL^−1^ cycloheximide and 50* *µCie of ^14^C-acetate were added simultaneously to the cells. After 7* *min of vigorous shaking cells were mixed with 35* *mL of cold TAP medium supplemented with 40* *mM acetate and immediately spun down. Cell pellets were afterwards washed in 5* *mM Hepes buffer supplemented with EDTA-free protease inhibitors mix (Roche), resuspended in 0.1* *M DTT, 0.2* *M Na_2_CO_3_, and flash-frozen. Prior to denaturation, samples were suspended 1:1 in 5% SDS, 20% sucrose solution, boiled for 1* *min, then spun down at 12.000* *g for 15* *min. The supernatant was subsequently loaded on urea 12–18% polyacrylamide gradient gels using in house-built gel system. Samples from pulse labeling experiments relying on ^14^C incorporation in the alga are loaded based on radioactivity incorporation rather than equal protein amount and rates of synthesis are estimated by comparison to labeling intensity of unrelated chloroplast translates. After migration, gels were stained with Coomassie Blue, dried, and exposed to an autoradiography screen for at least 1* *month. Phosphorescence signal was measured using a Typhoon FLA 9500 phosphorimager (GE Healthcare).

### Immunochase experiments

Exponentially growing Chlamydomonas cells were treated with chloramphenicol at a final concentration of 500* *µg/mL. For Figure 1D, 30* *mL aliquots were removed from a 200* *mL culture along the chase at the initial time-point of inhibitor addition and after 1, 2, and 4* *h, and were submitted to protein extraction followed by separation on 12% SDS-PAGE. Samples from different time-points were loaded on an equal chlorophyll basis, as by Kuras and Wollman (1994). For Figure 5C, a volume of 800* *mL of culture was separated into four erlens of 200* *mL to which CAP was added. At the initial time-point and after 2, 4, and 6* *h, soluble proteins were prepared from 200* *mL of culture and separated under native conditions as specified below (colorless native CN--PAGE).

### Protein analysis

*Protein electrophoresis* in denaturing conditions was performed according to the modified Laemmli protocol (Laemmli, 1970). For protein loading of “whole cell” samples, chlorophyll fluorescence was used for quantification as in (Kuras and Wollman, 1994). Samples were suspended 1:1 in 5% SDS, 20% sucrose solution and boiled for 1* *min, then spun down at 12.000* g* for 15* *min to remove insoluble material and subsequently analyzed using 12% (in-house gels) or 8–16% SDS-polyacrylamide gels (Bio-Rad).

*CN electrophoresis* (CN-PAGE) was done according to a modified Schägger protocol (Wittig and Schagger, 2005). Cell pellets from 200* *mL of Chlamydomonas culture were resuspended in an extraction buffer (20* *mM HEPES pH* *7.5, 20* *mM KCl, 10% glycerol, 2× EDTA-free protease inhibitor mix (ROCHE)), and broken using a French press apparatus at 6.000* *psi. The soluble fraction was collected after centrifugation at 267.000* *rcf at 4°C for 25* *min and concentrated using Amicon Ultra centrifugation units with a 30* *kDa cutoff (Millipore). Protein concentrations were measured colorimetrically by Bradford assay (Bradford, 1976) using QuickStart Bradford Dye reagent (Bio-Rad). An amount of 70* *µg of protein was loaded on MiniProtean 4–16% gradient gels (Invitrogen), as well as a native protein marker (NativeMark unstained Protein standard, Life Technologies). Migration was undertaken at 4°C at constant voltage of 60* *V for 1* *h than 120* *V. Native gels used for immunoblotting were first incubated 1 hour in 2% SDS, 0.67% β-mercaptoethanol prior to their transfer on nitrocellulose membranes (2* *h 30 at 1* *mA/cm^2^). CN-PAGE analyses were reproduced at least twice, except for the one displayed in Figure 6B.

*Coimmunoprecipitation* was performed using 400* *μg of soluble extracts, prepared as specified in the previous section (NEB buffer: Hepes-KOH pH 7.5 20* *mM, KCl 100* *mM, glycerol 10%), which were incubated for 30* *min at 4°C with 50* *μL of ferrimagnetic StrepTactin beads (MagStrep Type3 XT beads, IBA LifeSciences) preincubated in 0.1* *M Tris pH 8.0, 150* *mM NaCl, with regular mixing. A fraction of soluble extract (1/8 of the initial amount) was set aside to evaluate the protein composition of the input fraction. A magnetic stand was used to clear the beads from the supernatant. The unbound extract was then removed and an equivalent fraction (volume wise) set aside. Then, 3× Laemmli loading buffer was added to both the input and unbound fraction. Beads were washed three times in wash buffer (0.1* *M Tris pH 8.0, 150* *mM NaCl supplemented with protease inhibitors), and then resuspended in 1× Laemmli loading Buffer. Equivalent fractions of the input, unbound and eluted fractions were denatured for 1* *min at 100°C, cleared by centrifugation at 13.000 rpm at 4°C, and separated on a 10% acrylamide-urea 8M-SDS gel, followed by blotting and immunodecoration as specified in the next section.

For *immunoblot analysis*, proteins were transferred onto nitrocellulose membranes (0.1* *µ pore size, Amersham Protran). The membranes were blocked with 5% (w/v) skim milk in a phosphate-buffered saline (PBS) solution plus 0.1% (w/v) Tween 20 (Sigma-Aldrich) (PBST). The target proteins were immunodecorated by overnight incubation at 4°C with primary antibodies. Membranes were washed three times for 10* *min with PBST, and then incubated with horseradish peroxidase-conjugated anti-rabbit IgG antibodies (Catalogue number: W4011, Promega) at a dilution ratio of 1:20.000, followed by three additional washes in PBST. Primary antibodies used in this study are all polyclonal antibodies raised in rabbits. They were directed against spinach Rubisco whole holoenzyme (kindly provided by Dr. Spencer Whitney, used at a dilution of 1:40.000 to 1:80.000 ), Chlamydomonas Cpn60α/β1 (kind gift of Michael Schroda, used at a dilution of 1:2.000) and against the Chlamydomonas PSI subunit PsaD (kindly provided by Dr. Yuichiro Takahashi, and used at a dilution of 1:10.000). Antibody against cyt *f* (PetA; used at a dilution ratio of 1:100.000) is described by Kuras and Wollman (1994). For RAF1 polyclonal antibody production, part of the *C. reinhardtii* RAF1 gene (Cre06.g308450, underlined part in Supplemental Figure S3A) was expressed in *E. coli* using a codon-adapted synthetic cDNA (Genscript, Piscataway, NJ, USA). The recombinant protein was purified using GST-tag affinity and used directly as an antigen in rabbits (Genscript, Piscataway, NJ, USA). The resulting antiserum was used at a dilution of 1:30.000. Immuno-reactive proteins were detected with Clarity One detection reagent (Bio-Rad) and visualized using the ChemiDoc XRS+ System (Bio-Rad). As a rule, immunoblots were repeated at least twice, using no less than two independent transformants or mutant strains for each genetic background, out of which one is shown in the final figures.

### Accession numbers

Sequence data from genes discussed in this manuscript can be found on Phytozome and in the National Center for Biotechnology Information Database under the following gene identifier and accession numbers respectively: *rbcL* (ASF83644.1), *RBCS1* (Cre02.g120100, XP_001702409), *RBCS2* (Cre02.g120150, XP_001702408.1), *RAF1* (Cre06.g308450, PNW83144.1), *MRL1* (Cre06.g298300, PNW82886.1).

## Supplemental data

The following materials are available in the online version of this article.

Supplemental Figure S1. LSU stability and estimated half-life in different Rubisco mutants.

Supplemental Figure S2. Truncated LSU does not accumulate.

Supplemental Figure S3. Anti-RAF1 antibody.

Supplemental Figure S4. Led spectra used for Chlamydomonas growth.

Supplemental Table S1. Summary of transformation experiments.

Supplemental Table S2. List of primers.

## Supplementary Material

koab061_Supplementary_Data

## Notes

W.W., F.A.W., and K.W. designed the research; W.W., E.T., and K.W. performed research; W.W., F.A.W., and K.W. analyzed data and wrote the article.

The author responsible for distribution of materials integral to the findings presented in this article in accordance with the policy described in the Instructions for Authors (https://academic.oup.com/plcell) is: Katia Wostrikoff (Katia.Wostrikoff@ibpc.fr).
