# Supplementary material for: The state of oligomerization of Rubisco controls the rate of synthesis of the Rubisco large subunit in Chlamydomonas reinhardtii
Source: Plant Cell. 2021 Feb 24;33(5):1706–27. doi: 10.1093/plcell/koab061 (PMC8254502; doi:10.1093/plcell/koab061)
Supplement: koab061_Supplementary_Data [file koab061_supplementary_data.zip › tpc.00880.2020-s01.pdf]

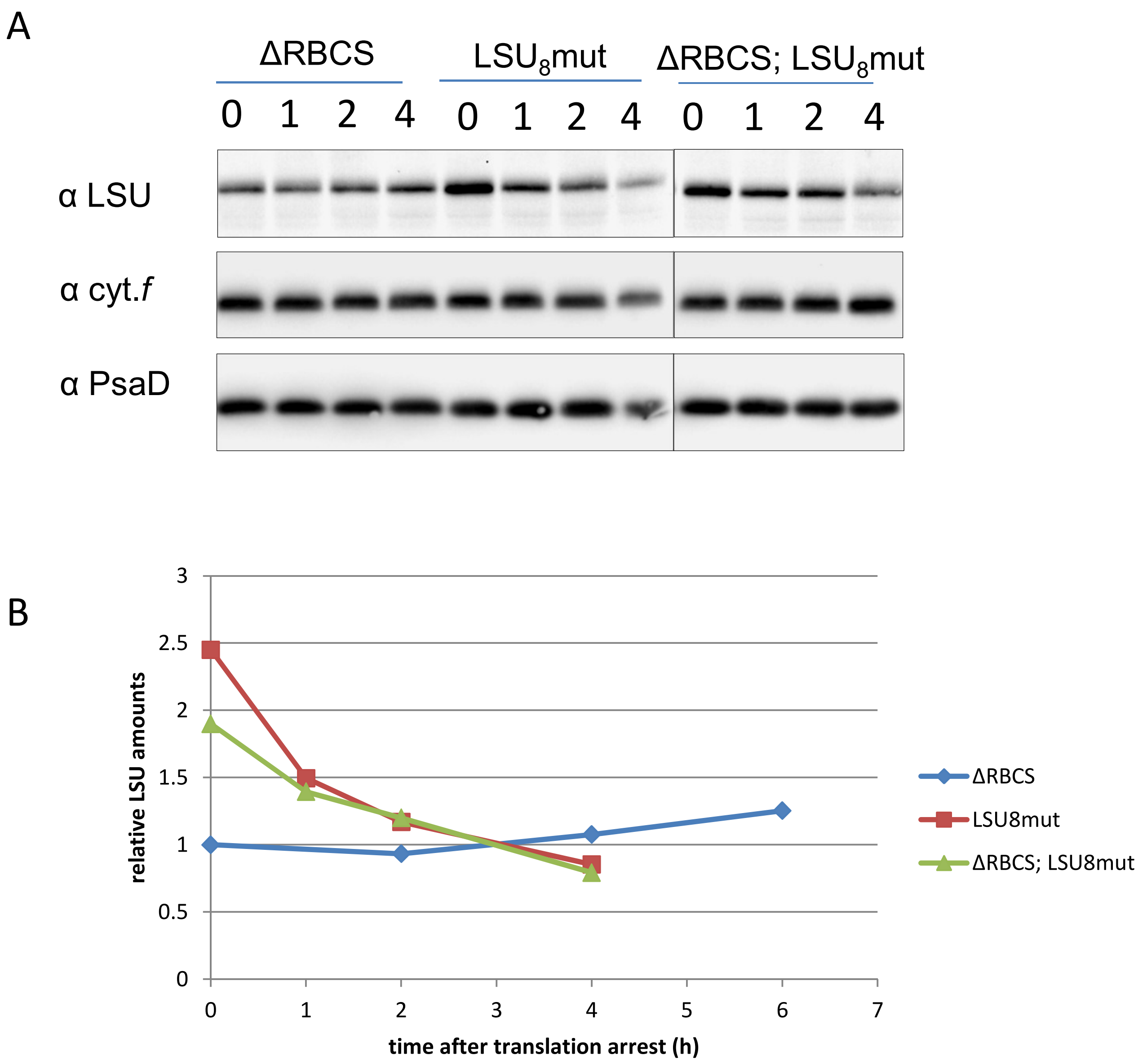

Supplemental Figure S1. LSU stability and half-life in different Rubisco mutants.
Supports Fig.1 and Fig. 9
(A) LSU stability assayed by immunochase over 4 hours after chloroplast synthesis arrest by chloramphenicol (CAP) addition in the LSU_8mut and ΔRBCS; LSU_8mut mutants and compared to ΔRBCS. LSU is detected with the anti-Rubisco antibody, cytochrome *f* and PsaD are used as loading controls. Note that the membrane immunodecorated with Rubisco antibody was exposed for a longer time compared to those exposed to the cytochrome *f* and PsaD antibodies in order to detect LSU, whose level drops to about 1-2 % in these strains. The right part of the figure separated by a line, comes from the same gel, was exposed using the same settings as the rest of the figure, but was not initially next to the samples of the LSU_8mut strains and moved for increased clarity.

(B) LSU degradation rate in the ΔRBCS and LSU_8mut strains, as calculated from densitometric signals obtained from relative LSU accumulation plotted over time, in immunochase experiments in absence of chloroplast synthesis, from gels presented in A. LSU half-life calculated over the first hour of chase is estimated to 30 minutes for LSU_8mut.

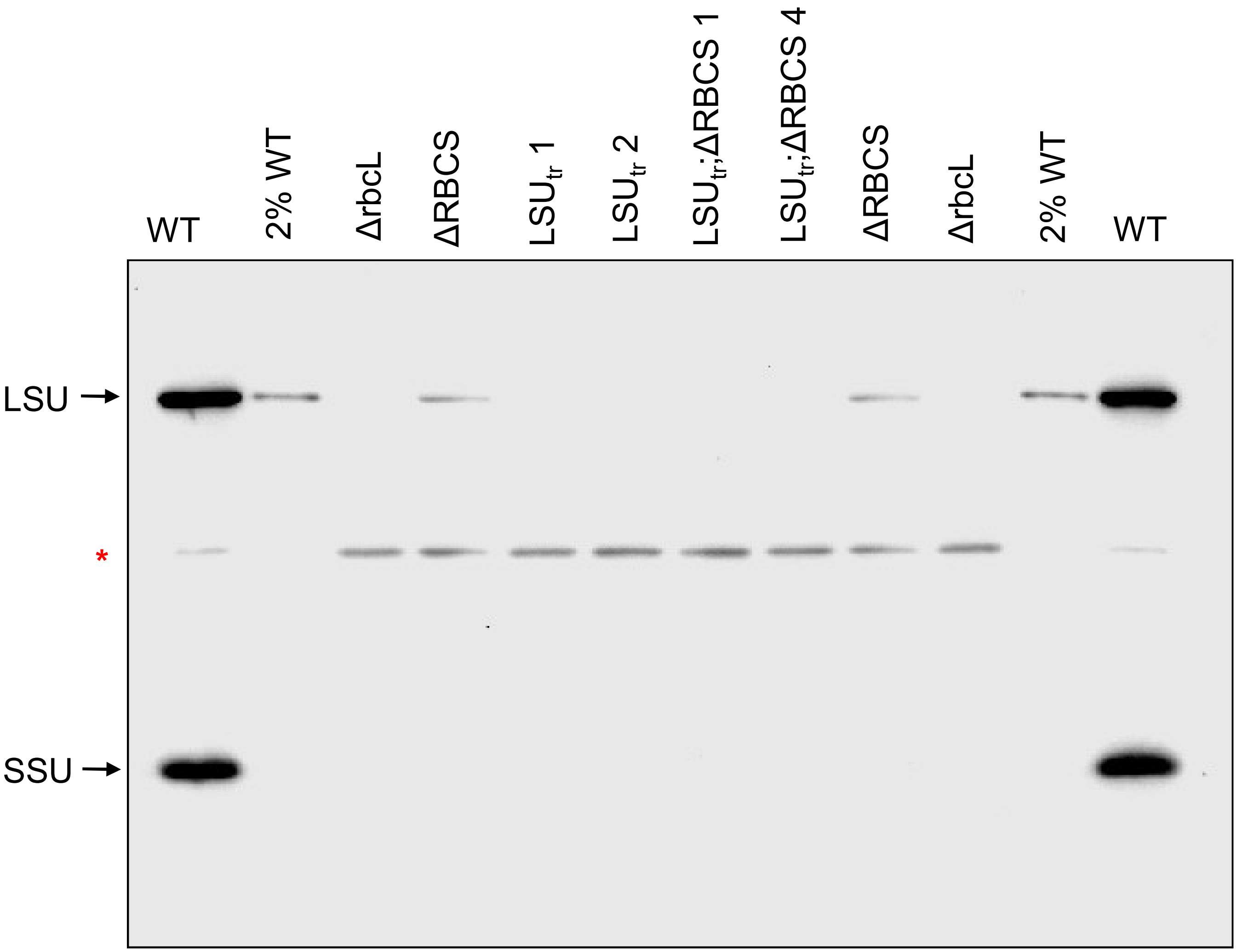

Supplemental Figure S2. Truncated LSU does not accumulate.

Supports Fig.4

Immunoblot showing accumulation of Rubisco subunits in transformants bearing a truncation within the *rbcL* gene (LSUtr), associated or not to the ΔRBCS mutation, and compared to the wild-type, ΔrbcL and ΔRBCS strains. A red asterisk marks the migration of a cross-reacting signal.

A

```
>CrRAF1
MKLTSSKSATSCRSSRRSSVAVHARFGRDSGLILGPNGPNNNPGNNNQKMKEQRLILPGQQQRGAPGGGRIVFPEKQGG
SGKPGGASMDNFLPDDSTLGLVGESLVGGPATLNKYRPPAGFMNENLPEDAYSSMDPQEMLNKLRARAGHWHELAKLMA
PLNSSGYSSSAIDELTGITPLEQSKWVVAATVYESVKASPAVSPDTLRHFNQGGEELLHPFRFLSAERRVSAAQYIAEQ
NLDPPMCEILARSMKEYERRPTERVGFTDHPADCLAFKYLRDAIECRKREEAVQKVEQGLSVALSDGARQRLYELLAES
PEEADTGSGVSASLVTLRLNPEELGVRPVAVLGELGHATIDDLQAAPRASQGGAFGMFTIEPADADASAGSSSAASTSG
RRASAAASSWVALPMWRALAMARQPVGLMLKDCAAVQAVLAGSKAKTDEDKKRLSGPGMIVADKAPATWQEAGLDENAW
YLAQPEGARAIQLVDGRRAKAQELDKRGALCGVVLFLARPPVKETEDRGHNLLQV
```

B

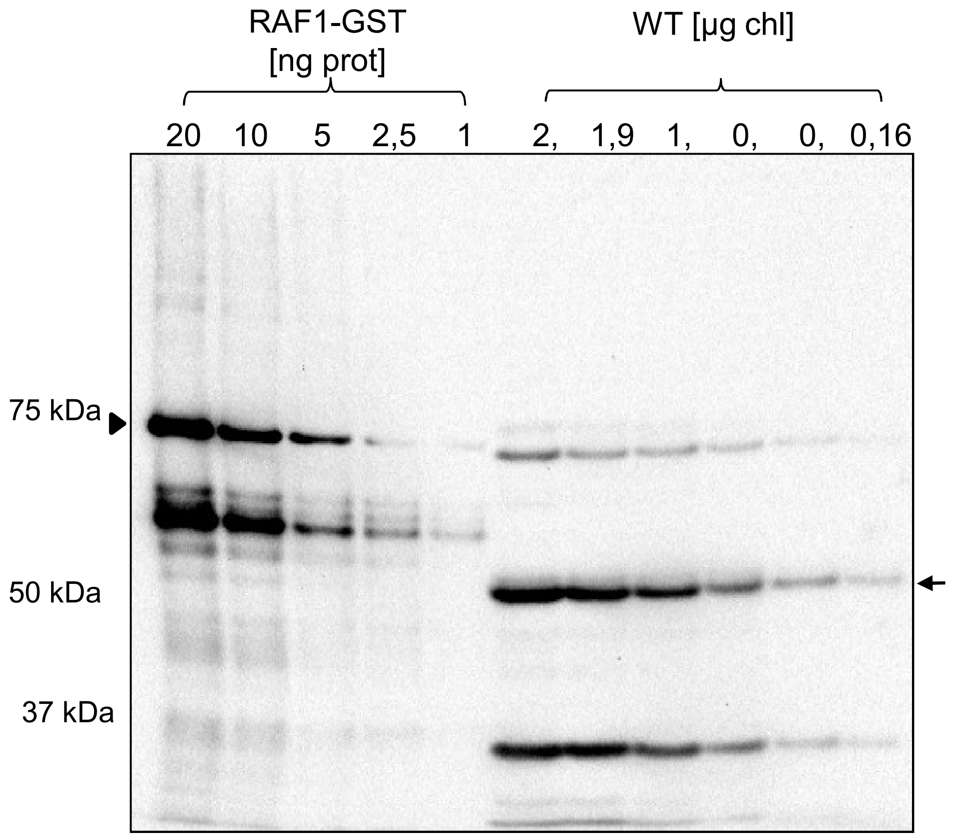

Supplemental Figure *S*3. Anti-RAF1 antibody.

Supports Fig. 5, Fig. 6 and Fig. 7

(A) Updated gene model for CrRAF1 (Cre06.g308450), extended at its N-terminus to an upstream in-frame Methionine compared to the predicted initiator Methionine (red) from Chlamydomonas v5.6 gene model. This updated gene model of 529 aa is covered by EST, predicted to be chloroplast-localized by the Predalgo localization program, and the detected RAF1 size corresponds to the predicted size minus the predicted signal sequence. The underlined sequence was used for recombinant expression in *E. coli* and antibody generation.

(B) Immunoblot showing reactivity of the raised α-RAF1 antibody. Dilutions of purified GST-tagged recombinant RAF1 protein (85% purity) were compared to dilutions of WT whole cell extracts. The positions of recombinant GST-tagged RAF1 expected at 75kDa (50 kDa RAF1 + 25 kDa GST-tag) and of Chlamydomonas RAF1 (expected at around 50kDa) are respectively indicated by a triangle and an arrow.

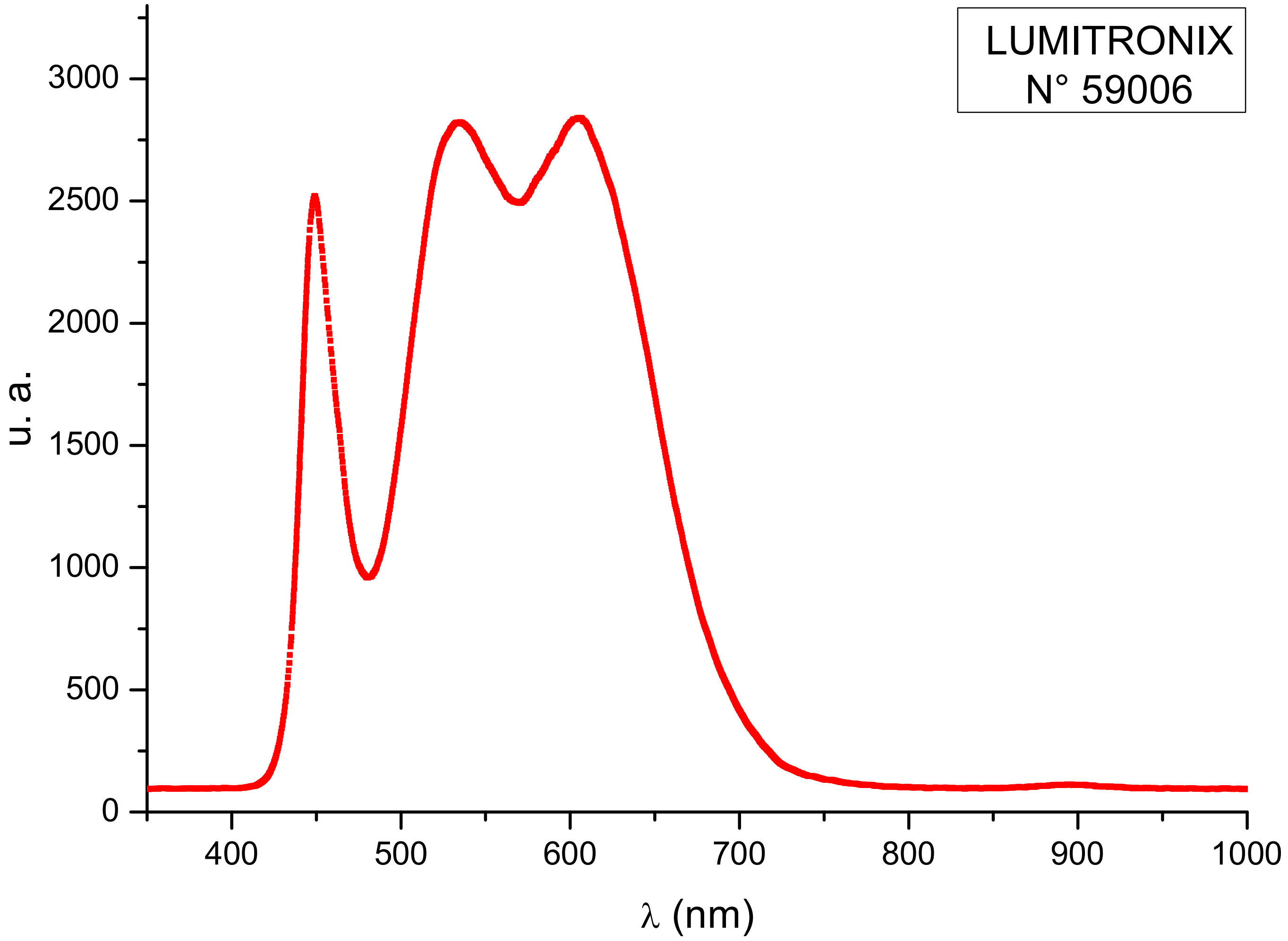

Supplemental Figure S4. Emission spectrum of the white led used to grow *C. reinhardtii*. Measured with a S2000 FiberOptics spectrophotometer (Ocean Optics, Inc).

Supplemental Table S1. Summary of transformation experiments.

Transformed strains	Recipient strains	Plasmid used	Selection	Ref
psaA 5'UTR-rbcL	ΔR T1.3 mt+	paAR	phototrophy	this work
rbcL 5'UTR-petA	WT mt+	pRFFFiK	Spectinomycin resistance	(Johnson et al., 2010)
ΔRBCS; rbcL 5'UTR-petA	Cal13.5A mt+	pRFFFiK	Spectinomycin resistance	This work
LSU_{tr}	ΔR T1.3 mt+	pLS_{tr}	Spectinomycin resistance	This work
ΔRBCS;LSU_{tr}	Cal13.5A mt+	pLS_{tr}	Spectinomycin resistance	This work
LSU_{tr};5'UTRrbcL:petA	RJ24 mt+ (rbcL 5'UTR-*petA* outcrossed to WT	pLS_{tr}	Spectinomycin resistance	This work
ΔRBCS;LSU_{tr};5'UTRrbcL:petA	RCaldK5 mt+(ΔRBCS; rbcL 5'UTR-petA)	pLS_{tr}	Spectinomycin resistance	This work
LSU_2mut	ΔR T1.3 mt+	pLS_2mut	Spectinomycin resistance	This work
LSU_8mut	ΔR T1.3 mt+	pLS ARD	Spectinomycin resistance	This work
ΔRBCS;LSU_8mut	Cal13.5A mt+	pLS ARD	Spectinomycin resistance	This work
ΔRBCS;5'UTR*rbcL*:*petA;*LSU_8mut	RCaldK5 mt+(ΔRBCS; rbcL 5'UTR-petA)	pLS ARD	Spectinomycin resistance	This work
ΔRBCS;RAF1Strep-TG	Cal13.1B mt-	pJHL-RAF1S	Paromomycin resistance	This work

- All recipient strains are sensitive to spectinomycin (Sp^S) and paromomycin.
- The ΔR T1.3 and RCaldK5 (ΔRBCS; rbcL 5'UTR-petA) strains were initially selected for spectinomycin resistance due to the presence of the recycling *aadA* cassette. After excision of the cassette according to (Fischer et al., 1996), the strains became Sp^S and were used as a recipient strain for a second round of transformation based on selection for spectinomycin resistance.

Supplemental references:

Fischer, N., Stampacchia, O., Redding, K., and Rochaix, J.-D. (1996). Selectable marker recycling in the chloroplast. M.G.G. 251, 373-380.

Johnson, X., Wostrikoff, K., Finazzi, G., Kuras, R., Schwarz, C., Bujaldon, S., Nickelsen, J., Stern, D.B., Wollman, F.A., and Vallon, O. (2010). MRL1, a conserved Pentatricopeptide repeat protein, is required for stabilization of rbcL mRNA in Chlamydomonas and Arabidopsis. The Plant Cell 22, 234-248.

Supplemental Table S2. Primer list.

Primer name	sequence
IP-R15 lin.F	CGTTTCCTTTTCGTTGCTGAAGC
IP-R15 lin.R	AGGTGGAATACGAAGGTCTTCAAG
IP- LS-A143.F	CTTCGTATTCCACCTtggTACGTTAAAACATTCGTA
IP-LS-R215D216.R	AACGAAAAGGAAACGtgCAgcCCAACGCATGAA
atpB Pst.F	gcg<u>ctgcag</u>CTATTAGTAAAGCTGCTTCATT
atpB Spe.R	tcg<u>actagt</u>TCACACTCTTATTATTTACTCGCACGT
IP-R15 E109.R	GAATAAGTCGATTGGGTAAGCTACG
IP-R15R253 Pst.F	gctGTATGTGCTAAAGAATTAGGTG
IP-LSE109A Bam.F	CCAATCGACTTATTCGctGAA<u>GGaTCc</u>GTAACTA
IP-LS R253A P.R	TTTAGCACATACaGCAgcTTTCATCATTTCTTCACAA
PsaAProm.F	cacgtgCTTTTACGAATACACATATGG
psaAProm-rbcL.R	AGTTTCTGTTTGTGGAACCATGGATTTCTCCTTATAATAAC
psaAPromRbcL.F	GTTATTATAAGGAGAAATCCATGGTTCCACAAACAGAAACT
RbcL EcoNI.R	CGACCGTAGTTTTTAGCTGAA
IP-PsaAProm.F	GAGAGGAGTGAACAGTCACGTGCTTTTAC
IP-RbcL EcoNI.R	CATAAACTGCACGACCGTAGTTTTTAGCTGAAAGAC
IP-R15 BseRI.R	ACTGTTCACTCCTCTCCAATATAGTAG
IP-R15 EcoNI.F2	GTCGTGCAGTTTATGAATGTTTAC
LSmutA143W.F	TGAAGACCTTCGTATTCCACCTTGG
dRLS.R1	GGAACACCTGGTTGTGGAGT
dRLS.F	AAGTTTATGACGCCGATTGC
dRLS.R2	AGAGTACCACCACCGAACTG
LSA143wt.F	TGAAGACCTTCGTATTCCACCTGCT
LSmutD216A.R	GCTTCAGCAACGAAAAGGAAACGTG
LSD216wt.R	GCTTCAGCAACGAAAAGGAAACGGTC
LSmutE109A.F	AGCTTACCCAATCGACTTATTCGCT
LSE109wt.F	CGTAGCTTACCCAATCGACTTATTCGAA
LSmutR253A.R	CTAATTCTTTAGCACATACAGCAGC
LSR253wt.R	TAATTCTTTAGCACATACTGCACG
CrRbcLProm.F2	TCGACTGATAAGACAAGTACAT
CrRbcL.R3	CATAAACATCATGAAAAATAAAAATTAAAG
CrRbcL EcoNI.R	CGACCGTAGTTTTTAGCTGAA
CrRAF1.F2	CCTGCGGCTTTCTTCTTTGT
PsaD.R	CTGTGGCTAATTGACCGTGGG

Modified sequences compared to the endogenous sequence are shown in lowercase letters, restriction sites are underlined.
